# Exploring the cost-effectiveness of a Dutch collaborative stepped care intervention for the treatment of depression and/or anxiety when adapted to the Australian context: a model-based cost-utility analysis

**DOI:** 10.1017/S2045796021000470

**Published:** 2021-08-25

**Authors:** Y. Y. Lee, M. G. Harris, H. A. Whiteford, S. K. Davidson, M. L. Chatterton, E. A. Stockings, C. Mihalopoulos

**Affiliations:** 1 Faculty of Health, Deakin University, Deakin Health Economics, Institute for Health Transformation, School for Health and Social Development, Geelong, Australia; 2 School of Public Health, The University of Queensland, Brisbane, Queensland, Australia; 3 Policy and Epidemiology Group, Queensland Centre for Mental Health Research, Brisbane, Queensland, Australia; 4 Institute for Health Metrics and Evaluation, University of Washington, Seattle, Washington, USA; 5 Department of General Practice, University of Melbourne, Parkville, Victoria, Australia; 6 National Drug and Alcohol Research Centre, University of New South Wales, Randwick, New South Wales, Australia

**Keywords:** Anxiety disorders, collaborative care, cost-effectiveness analysis, depressive disorder, economic evaluation, general practice, stepped care

## Abstract

**Aims:**

Depression and anxiety are among the most common mental health conditions treated in primary care. They frequently co-occur and involve recommended treatments that overlap. Evidence from randomised controlled trials (RCTs) shows specific stepped care interventions to be cost-effective in improving symptom remission. However, most RCTs have focused on either depression or anxiety, which limits their generalisability to routine primary care settings. This study aimed to evaluate the cost-effectiveness of a collaborative stepped care (CSC) intervention to treat depression and/or anxiety among adults in Australian primary care settings.

**Method:**

A quasi-decision tree model was developed to evaluate the cost-effectiveness of a CSC intervention relative to care-as-usual (CAU). The model adapted a CSC intervention described in a previous Dutch RCT to the Australian context. This 8-month, cluster RCT recruited patients with depression and/or anxiety (*n* = 158) from 30 primary care clinics in the Netherlands. The CSC intervention involved two steps: (1) guided self-help with a nurse at a primary care clinic; and (2) referral to specialised mental healthcare. The cost-effectiveness model adopted a health sector perspective and synthesised data from two main sources: RCT data on intervention pathways, remission probabilities and healthcare service utilisation; and Australia-specific data on demography, epidemiology and unit costs from external sources. Incremental costs and incremental health outcomes were estimated across a 1-year time horizon. Health outcomes were measured as disability-adjusted life years (DALYs) due to remitted cases of depression and/or anxiety. Incremental cost-effectiveness ratios (ICERs) were measured in 2019 Australian dollars (A$) per DALY averted. Uncertainty and sensitivity analyses were performed to test the robustness of cost-effectiveness findings.

**Result:**

The CSC intervention had a high probability (99.6%) of being cost-effective relative to CAU. The resulting ICER (A$5207/DALY; 95% uncertainty interval: *dominant* to 25 345) fell below the willingness-to-pay threshold of A$50 000/DALY. ICERs were robust to changes in model parameters and assumptions.

**Conclusions:**

This study found that a Dutch CSC intervention, with nurse-delivered guided self-help treatment as a first step, could potentially be cost-effective in treating depression and/or anxiety if transferred to the Australian primary care context. However, adaptations may be required to ensure feasibility and acceptability in the Australian healthcare context. In addition, further evidence is needed to verify the real-world cost-effectiveness of the CSC intervention when implemented in routine practice and to evaluate its effectiveness/cost-effectiveness when compared to other viable stepped care interventions for the treatment of depression and/or anxiety.

## Introduction

Mental health policy makers are increasingly considering a stepped care approach to organising and delivering accessible, effective services to people with depression and/or anxiety (Richards *et al*., 2012; van Straten *et al*., 2015; Ho *et al*., 2016). The stepped care approach involves administering evidence-based treatment interventions of increasing intensity to patients across several steps and is based on two core principles (Bower and Gilbody, 2005). The first principle is that patients begin with the ‘least restrictive’ (i.e. lowest intensity) intervention appropriate to their level of need. The second principle involves ‘self-correction’, whereby patients are referred to a higher intensity intervention if an inadequate treatment response is identified during routine monitoring of patient outcomes. The intensity of an intervention is often characterised by the amount of therapist time involved in treatment provision (or the addition of medication) (Bower and Gilbody, 2005; van Straten *et al*., 2015). Stepped care differs from standard approaches to mental healthcare which are commonly criticised for generating high levels of treatment misallocation – i.e. over-treatment of mild/moderate cases and under-treatment of moderate/severe cases. Stepped care is designed to reduce treatment misallocation by encouraging patients with lower levels of need to access low-cost, low-intensity treatments; thereby freeing up high-cost, high-intensity treatments for patients with greater needs (Bower and Gilbody, 2005).

There are many types of stepped care models that operationalise the stepped care approach across a range of settings. Randomised controlled trials (RCTs) have found that stepped care models involving a progressive increase in treatment intensity can be more efficacious than care-as-usual (CAU) when treating depression and/or anxiety among adults (van Straten *et al*., 2015; Ho *et al*., 2016). However, there is extensive variation between models with respect to the: number and duration of steps; types of treatment provided at each step; and types of healthcare providers involved (van Straten *et al*., 2015). To date, a handful of economic evaluations have evaluated stepped care models to treat either depression only (Meeuwissen *et al*., 2019; Yan *et al*., 2019; Brettschneider *et al*., 2020) or anxiety only (Goorden *et al*., 2014; Stiles *et al*., 2019) in adults, but none have focused on depression and/or anxiety together. This is important because depression and anxiety frequently co-occur and involve similar, often overlapping, treatments (Burgess *et al*., 2009; NICE, 2009). These disorders are particularly common among patients presenting in the primary care setting where general practitioners (GPs) require clear, straightforward clinical guidance (Cohen, 2018).

The Australian Federal government has endorsed the implementation of a stepped care approach to enhance mental health service delivery across the national system of Primary Health Networks (PHNs) – i.e. government agencies responsible for delivering integrated primary healthcare services within defined geographical regions across Australia (Department of Health, 2020). One Australian study has previously examined the cost-effectiveness of a stepped care model, incorporating low-intensity treatment as a first step, to treat anxiety only (Stiles *et al*., 2019). However, no published study has examined the role of a stepped care model for the treatment of depression and/or anxiety in Australia. This study will evaluate the cost-effectiveness of adapting a stepped care model developed in the Netherlands for the treatment of depression and/or anxiety across the Australian adult population.

## Methods

### Analytic approach

A decision-analytic model was developed to evaluate the cost-effectiveness of stepped care to treat adult depression and/or anxiety (hereafter referred to as depression/anxiety). Previous systematic reviews were used to identify RCTs of stepped care models for the treatment of depression/anxiety that had evidence of effectiveness and could be feasibly implemented in Australia. In-scope RCTs informed the design of a cost-effectiveness model based on the Assessing Cost-Effectiveness (ACE) approach – i.e. a framework previously developed to evaluate healthcare interventions in the Australian context (Vos *et al*., 2010). A health sector perspective was adopted to analyse the costs and health outcomes accruing to patients, healthcare providers and the government as a third-party payer. Cost offsets (i.e. treatment costs averted due to fewer cases of depression/anxiety following treatment) were included in the base case analysis; while productivity impacts (i.e. presenteeism and/or absenteeism due to poor health) were excluded as they occur beyond the health sector. Patient time and travel costs were excluded from the base case analysis, but included in a sensitivity analysis.

The cost-effectiveness model was developed using Microsoft Excel 2016. It comprised a cost-utility analysis that measured health outcomes using the disability-adjusted life year (DALY) metric and costs in 2019 Australian dollars (A$). The model calculated incremental cost-effectiveness ratios (ICERs) which were evaluated with respect to an Australian willingness-to-pay (WTP) threshold of A$50 000 per DALY averted (Vos *et al*., 2010). Discounting was not necessary as the model employed a 1-year time horizon with 2019 as the reference year. Australian health price deflators were used to convert costs into 2019 Australian dollars (AIHW, 2020). Study findings have been reported in accordance with Consolidated Health Economic Evaluation Reporting Standards (CHEERS) (see online Supplementary Table S1).

### Evidence of effectiveness

RCTs on the effectiveness of stepped care treatment for adult depression/anxiety were identified from two previous systematic reviews (van Straten *et al*., 2015; Ho *et al*., 2016); and a search of PubMed and Google Scholar to find additional studies up to December 2020. In-scope RCTs for the cost-effectiveness model included those which: analysed stepped care treatment for depression/anxiety among adults; involved a progressive increase in treatment intensity; contained sufficient data to inform cost-effectiveness modelling; universally targeted all patients with depression/anxiety; was generalisable to the Australian setting; and involved a CAU control arm reflective of services currently offered in Australia. RCTs involving the treatment of depression only or anxiety only were excluded. A total of 12 RCTs examined stepped care models involving a progressive increase in treatment intensity. Of these, 11 were excluded with reasons (see online Supplementary Text S2).

The remaining in-scope RCT by Oosterbaan *et al*. (2013) evaluated the effectiveness of collaborative stepped care (CSC) for the treatment of depression/anxiety *v.* CAU over an 8-month period, among 163 adult patients attending primary care clinics in the Netherlands (see Table 1 for sample characteristics). This stepped care model incorporated a collaborative care approach which integrated health professionals from a range of disciplines (e.g. nurses, psychologists and psychiatrists) to help liaise with patients and assist GPs in providing evidence-based treatment (Katon *et al*., 1999). The CSC intervention involved two steps: (1) guided self-help with a mental health nurse at a local GP clinic for patients with a depressive/anxiety disorder of mild-to-moderate severity – plus antidepressants for those with a moderate disorder; and (2) treatment with antidepressants, psychotherapy or a combination of both at an outpatient specialised mental healthcare clinic for patients who do not remit 4 months after commencing the first step. Patients with a severe disorder at baseline were automatically assigned to receive treatment in the second step. Patients who were actively suicidal or had psychotic symptoms were referred to tertiary clinics for intensive treatment. Patients with comorbid depression/anxiety were assigned a primary diagnosis based on the most problematic disorder. The CAU comparator encompassed treatment options that are normally available to patients treated by GPs in the Netherlands: no treatment; prescription of antidepressants; referral to a mental health nurse at a GP clinic; or referral to a specialised mental healthcare centre (or other professional) where the patient received treatment with antidepressants, psychotherapy or both. The primary clinical outcome was remission – defined as a score <3 (out of 7) on the Clinical Global Impression Severity Scale (CGI-S). Remission probabilities were assessed at 4 months (mid-test), 8 months (post-test) and 12 months (follow-up).

**Table 1.** Demographic and clinical characteristics of study participants in the randomised controlled trial by Oosterbaan *et al*. (2013)

Sample characteristics	CSC group (*n* *=* 94)	CAU group (*n* *=* 64)	*p* value
Demographic characteristics			
Age in years, mean (s.d.)	37.0 (11.8)	39.4 (12.4)	0.23
Female, *n* (%)	59 (62.8)	39 (60.9)	0.82
Higher education (college or university), *n* (%)	19 (20.2)	15 (23.4)	0.65
Employed, *n* (%)	73 (77.7)	43 (67.2)	0.13
Foreign, *n* (%)	5 (5.3)	6 (9.4)	0.33
Clinical characteristics			
Number of mental health diagnoses, mean (s.d.)	2.3 (1.2)	2.1 (1.1)	0.29
Duration of diagnosis in years, mean (s.d.)	3.4 (6.3)	3.2 (6.1)	0.83
Psychiatric history, *n* (%)	34 (36.2)	21 (32.8)	0.66
Severely ill^a^, *n* (%)	25 (26.6)	20 (31.3)	0.53
Main diagnosis^b^			
Any depressive disorder, *n* (%)	47 (50.0)	26 (40.6)	0.25
Any anxiety disorder, *n* (%)	29 (30.9)	28 (43.8)	0.10
Any stress-related disorder^c^, *n* (%)	18 (19.1)	10 (15.6)	0.57

CAU, care-as-usual; CSC, collaborative stepped care; *n*, sample size; s.d., standard deviation.

Data presented in the table above were adapted from a corresponding table in *Oosterbaan et al. (2013)*.

a Severely ill: at least markedly severe (score ⩾5) on seven-point Clinical Global Impression Severity Scale.

b Patients with comorbid depression, anxiety and/or stress-related disorder were assigned a primary diagnosis based on the most problematic disorder.

c The original randomised controlled trial by *Oosterbaan et al. (2013)* included an intervention pathway for people with a diagnosis of stress-related adjustment disorder. However, the number of patients allocated to the stress treatment program was too small to permit a separate analysis. It follows that the trial only reported outcomes for study participants allocated to the treatment programs for depression and anxiety. The cost-effectiveness model similarly focussed on intervention pathways for the treatment of depression and/or anxiety.

A quasi-decision tree model was developed using data from the RCT (Oosterbaan *et al*., 2013) to evaluate the incremental cost-effectiveness of the CSC intervention relative to the CAU comparator over a 1-year time horizon. The cost-effectiveness model was informed by: (1) RCT data on intervention pathways, remission probabilities and healthcare service utilisation; and (2) Australia-specific data on demography, epidemiology and unit costs which were synthesised from several external data sources (described in later sections). The model consequently evaluated whether the CSC intervention would be cost-effective if it were adapted to the Australian context (assuming remission probabilities observed in the RCT were replicable). It was also assumed that the CSC intervention was fully implemented such that it would operate under ‘steady-state’ conditions – i.e. trained staff and required infrastructure were readily available (Vos *et al*., 2010). Since the RCT reported separate remission probabilities for depression and anxiety, it was possible to design the cost-effectiveness model to report results when targeting treatment to those with: (1) depression and/or anxiety; (2) depression only; and (3) anxiety only.

### Eligible population

The eligible population was defined in accordance with the RCT (Oosterbaan *et al*., 2013) and extrapolated to the 2019 Australian population. It included all adults who: were aged 18 years and above; visited a GP clinic in the past year for mental health treatment; and have an in-scope depressive/anxiety disorder. In-scope depressive disorders encompassed major depressive disorder and dysthymia; while in-scope anxiety disorders comprised panic disorder, agoraphobia, social phobia and generalised anxiety disorder. The 2019 Australian population was based on data from the Australian Bureau of Statistics (ABS) (ABS, 2020). Data from the Global Burden of Disease Study 2019 (GBD 2019) were used to calculate the prevalence of depression/anxiety in the 2019 Australian population (IHME, 2021); after making several adjustments using data from the 2007 National Survey of Mental Health and Wellbeing (Slade *et al*., 2009) (see online Supplementary Table S3). Data on the severity of depression/anxiety were also obtained from GBD 2019 (IHME, 2021). The 2019 Australian population, adjusted prevalence estimates for depression/anxiety and severity distributions are shown in online Supplementary Table S4.

### Health outcomes modelling

Remission outcomes for the CSC intervention and CAU comparator (see online Supplementary Table S4) were measured as 4-month probabilities and reported separately for depression and anxiety at 4-, 8- and 12-month intervals (Oosterbaan *et al*., 2013). Remission probabilities were multiplied by the number of people in each age-sex cohort of the eligible population during each 4-month interval to estimate the total number of remitted and unremitted cases that occur over a 1-year period among those with depression/anxiety at baseline. Area-under-the-curve methods were used to calculate the total number of life years during which patients were either in the remitted or unremitted health state (Matthews *et al*., 1990). Matching GBD 2019 disability weights (see online Supplementary Table S4) were assigned to people in the remitted and unremitted health states to calculate total DALYs averted (IHME, 2021). The formula used to calculate DALYs averted was: (1 − DW) × LY, where DW is the disability weight; and LY is the total life years in a remitted/unremitted health state. It inverts the disability weight by transforming it from a measure of health loss to a measure of health gain (Musgrove and Fox-Rushby, 2006; Neumann *et al*., 2018); thus giving it a similar interpretation to utility weights which are used to calculate quality-adjusted life years (QALYs). Disorder-specific disability weights were adjusted to account for background morbidity due to other causes of disease and injury (see online Supplementary Text S5).

### Cost analysis

**Fig. 1.**
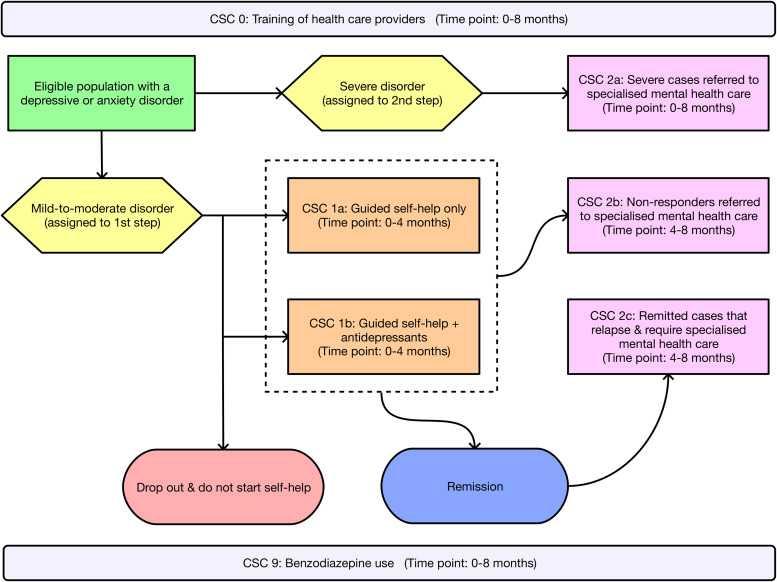
Intervention pathway for the collaborative stepped care (CSC) intervention. *Note*: The initial and ongoing training of healthcare providers (CSC 0) occurs over the course of the 8-month CSC intervention. Similarly, benzodiazepine use (CSC 9) occurs across a proportion of patients over the course of the 8-month CSC intervention.

**Fig. 2.**
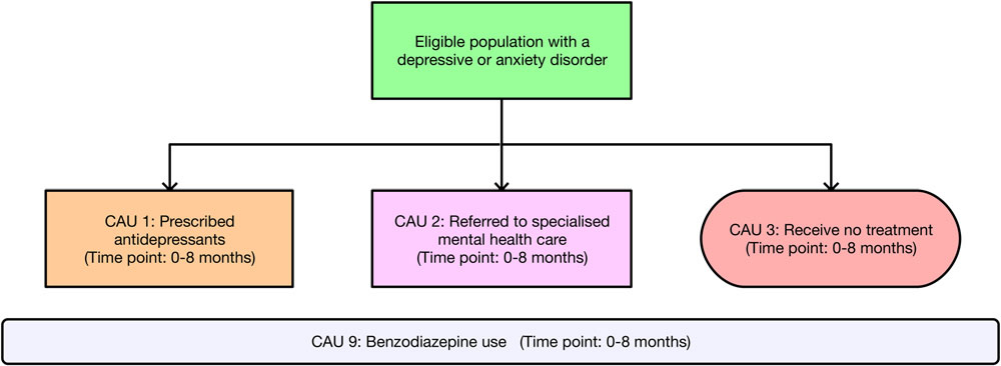
Intervention pathway for the care-as-usual (CAU) comparator. *Note*: Patients who drop out are included among those who receive no treatment (CAU 3). Benzodiazepine use (CAU 9) occurs across a proportion of patients over the course of the 8-month CAU comparator.

Intervention pathways described in the RCT (Oosterbaan *et al*., 2013) occur over an 8-month time period and are presented for the CSC intervention in Fig. 1 and the CAU comparator in Fig. 2. Costs for the CSC intervention pathway were grouped into four categories: the cost of training healthcare providers (CSC 0); the first step for mild disorders involving guided self-help only (CSC 1a); the first step for moderate disorders comprising guided self-help plus antidepressant medication (CSC 1b); and the second step involving specialised mental healthcare (CSC 2a, 2b and 2c). Cost categories in the CAU comparator pathway included treatment with antidepressants only (CAU 1); referral to specialised mental healthcare (CAU 2); and receiving no treatment (CAU 3). Over the 0–8 months of intervention delivery, GPs were free to prescribe benzodiazepines to all patients on top of existing treatments across both the CSC intervention (CSC 9) and CAU comparator (CAU 9). Relevant local data were used to adapt intervention pathways to the Australian context. In addition to intervention pathway costs, the annual treatment costs associated with unremitted cases of depression/anxiety (used to estimate cost offsets) were calculated for both the CSC intervention and CAU comparator. Methods describing the estimation of costs and local Australian context implementation for the CSC intervention and the CAU comparator are presented in online Supplementary Text S6. Cost analysis parameters and data are presented in online Supplementary Table S7.

### Cost-effectiveness analysis

The cost-effectiveness model used incremental analysis to compare costs and health outcomes between the CSC intervention and CAU comparator. Incremental intervention pathway costs comprised the difference in costs between the intervention and comparator pathways. Cost offsets due to avoided treatment costs involved the difference in treatment costs for unremitted cases of depression/anxiety between the intervention and comparator. Net incremental costs were estimated as the sum of incremental intervention pathway costs and cost offsets. Incremental health outcomes (i.e. incremental DALYs averted) comprised the difference in total DALYs averted between the intervention and comparator. ICERs were estimated by dividing net incremental costs by incremental DALYs averted.

### Uncertainty and sensitivity analyses

An uncertainty analysis was conducted to quantify the impact of input parameter uncertainty on the results of the cost-effectiveness model. The Ersatz program (version 1.31, Sunrise Beach, Australia; available at: http://www.epigear.com/) was used to perform Monte Carlo simulation with 3000 iterations and produce 95% uncertainty intervals (95% UI) around all model outputs. Uncertainty parameters are presented in online Supplementary Table S4 for health outcome modelling inputs and online Supplementary Table S7 for cost analysis inputs. Uncertainty analysis results were presented on a cost-effectiveness plane, where incremental DALYs averted are plotted on the *x*-axis; net incremental costs are plotted on the *y*-axis; and the origin (0, 0) represents the comparator. The cost-effectiveness plane is divided into four quadrants that each indicates how costly and effective the intervention is relative to the comparator. Cost-effectiveness acceptability curves were used to gauge the probability of the CSC intervention being cost-effective relative to different WTP thresholds.

A univariate sensitivity analysis was performed to test the robustness of final cost-effectiveness outcomes to ±10% changes around input parameter values; with results presented on a tornado plot. In addition, scenario analyses were conducted to evaluate the impact of excluding cost offsets (SA1); and including patient time and travel costs (SA2). Time and travel cost parameters are presented in online Supplementary Table S8.

## Results

**Fig. 3.**
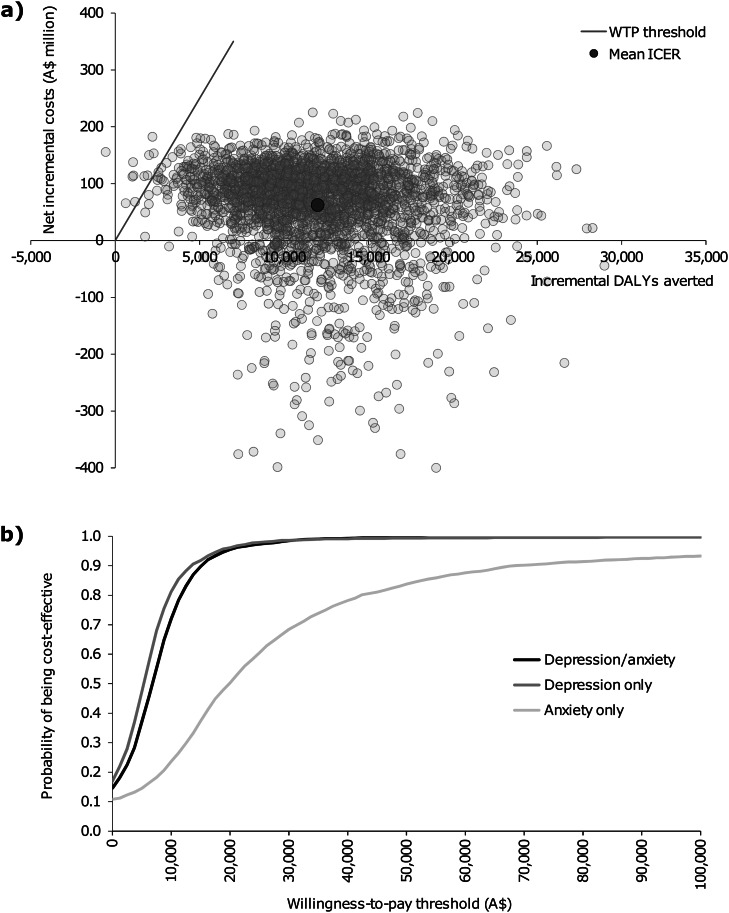
Graphs of the: (a) cost-effectiveness plane for the base case analysis involving depression and/or anxiety; and (b) cost-effectiveness acceptability curves. A$, Australian dollars; DALYs, disability-adjusted life years; ICER, incremental cost-effectiveness ratio; WTP, willingness-to-pay.

The CSC intervention was found to be cost-effective in treating depression/anxiety when compared with the CAU comparator. It produced an ICER of A$5207/DALY (95% UI: *dominant* to $25 345) which fell below the WTP threshold of A$50 000/DALY (see Table 2). An ICER of similar magnitude was produced when analysing depression only (A$3778/DALY; 95% UI: *dominant* to $23 570). When analysing anxiety only, wide uncertainty intervals were observed around the ICER (A$20 323/DALY; 95% UI: *dominant* to *dominated*). The CSC intervention had a 5.4% probability of being dominated, such that the cost of the CSC intervention exceeded the CAU comparator while simultaneously leading to poorer health outcomes. The cost-effectiveness plane for the analysis of depression/anxiety is presented in Fig. 3. The majority of uncertainty iterations (99.6%) were situated below the WTP threshold. Moreover, 14.6% of uncertainty iterations were situated in the south-east quadrant; indicating that the CSC intervention has a chance of producing cost-savings alongside positive health outcomes relative to the CAU comparator. Cost-effectiveness planes for analyses involving depression only and anxiety only are presented in online Supplementary Figs S9 and S10, respectively. The cost-effectiveness acceptability curves presented in Fig. 3 demonstrate that the CSC intervention had a high probability of cost-effectiveness when compared to a range of WTP thresholds below the designated WTP threshold of A$50 000/DALY.

**Table 2.** Cost-effectiveness results for the base case analysis

Output	Depression/anxiety (95% UI)	Depression only (95% UI)	Anxiety only (95% UI)
Net incremental costs (A$)	$62M (−$167M to $167M)	$38M (−$154M to $117M)	$41M (−$21M to $75M)
Incremental intervention pathway costs (A$)	$132M ($71M to $203M)	$92M ($48M to $145M)	$57M ($34M to $84M)
Cost offsets (A$)	−$70M (−$300M to −$6M)	−$54M (−$246M to −$4M)	−$16M (−$77M to $2M)
Incremental DALYs averted	11 991 (4564 to 20 863)	9987 (3060 to 18 534)	2004 (−423 to 4918)
Mean ICER (A$ per DALY averted)	$5207 (dominant to $25 345)	$3778 (dominant to $23 570)	$20 323 (dominant to dominated)

95% UI, 95% uncertainty interval; A$, Australian dollars; DALY, disability-adjusted life years; ICER, incremental cost-effectiveness ratio; M, million.

A dominant ICER indicates that the intervention costs less and is more effective than the comparator (i.e. South-East quadrant of the cost-effectiveness plane). Conversely, a dominated ICER indicates that the intervention costs more and is less effective than the comparator (i.e. North-West quadrant of the cost-effectiveness plane).

The results of the univariate sensitivity analysis involving depression/anxiety are presented in online Supplementary Fig. S11. Changes to remission outcomes for depression led to the greatest impact on resulting ICERs – i.e. a change >10%. The next largest impact involved the annual treatment cost per unremitted case of depression/anxiety (used to calculate cost offsets). Scenario analysis results are shown in online Supplementary Table S12. Despite higher ICERs being observed across every scenario, all ICERs remained below the WTP threshold of A$50 000/DALY.

## Discussion

### Summary of findings and comparison to previous studies

This exploratory analysis found that the Dutch CSC intervention had the potential to be cost-effective in treating depression/anxiety compared to the CAU comparator if adapted to the Australian context. These results were robust to changes implemented across scenario analyses. Even so, the univariate sensitivity analysis demonstrated that cost-effectiveness results were highly sensitive to changes to remission outcomes. Several international studies have examined the cost-effectiveness of alternative stepped care models incorporating low-intensity treatment as a first step for the treatment of depression only (Meeuwissen *et al*., 2019; Yan *et al*., 2019; Brettschneider *et al*., 2020) or anxiety only (Goorden *et al*., 2014; Stiles *et al*., 2019) among adult populations. All stepped care models in these studies were compared to CAU as a comparator. A trial-based cost-utility analysis from Canada observed that stepped care treatment for depression led to lower costs than CAU, despite the absence of significant differences between health outcomes (Yan *et al*., 2019). A model-based cost-utility analysis from the Netherlands found that stepped care treatment for depression was likely to be cost-effective relative to a €20 000 per QALY threshold (Meeuwissen *et al*., 2019). Conversely, a trial-based cost-utility analysis from Germany found no evidence that stepped care treatment for depression was cost-effective compared to CAU (Brettschneider *et al*., 2020). Stepped care treatment for anxiety was deemed cost-effective by an Australian model-based cost-utility analysis (Stiles *et al*., 2019) and a trial-based cost-utility analysis from the Netherlands (Goorden *et al*., 2014).

It is worth noting that the previous Australian study by Stiles *et al*. (2019) produced an ICER of A$3082/DALY (converted to 2019 Australian dollars); which was lower than the ICER observed by the present study when analysing stepped care treatment for anxiety only (i.e. A$20 323/DALY). These differences are partly explained by variations in the number and intensity of treatment steps. For example, the third and final step in Stiles *et al*. (2019) involved 6 months of pharmacotherapy with GP titration reviews (cf. the final step in the present study comprising outpatient specialised mental healthcare). Moreover, the previous Australian study excluded people with severe anxiety from the eligible population. Any comparison of findings between the present study and other economic evaluations should account for operational differences in underlying stepped care models.

### Implications of the findings

The results of this study suggest that a CSC intervention implemented in the Netherlands, which includes nurse-delivered guided self-help as a first step, is worth considering for adaptation and further testing by Australian PHNs due to its favourable cost-effectiveness profile. Even so, nurse-delivered guided self-help might be considered an intensive first-step treatment (in terms of therapist time) for patients with mild-to-moderate depression/anxiety. Other lower intensity interventions may be as appropriate for mild-to-moderate cases – e.g. unguided self-help, guided self-help delivered online and watchful waiting. Moreover, any prospective adaptation of the CSC intervention may encounter issues around the availability of appropriately trained mental health nurses and the acceptability to stakeholders of a nurse-led treatment model within GP clinics. Limited access to specialised mental healthcare may also be an issue for people living in rural/remote regions of Australia.

In Australia, government guidance on the wide-scale implementation of stepped care across the primary care setting has predominantly focussed on defining the types of care to be provided across different levels of mental illness severity (Department of Health, 2020). Comparatively little detail has been provided on: (1) how service providers might practically go about matching an initial treatment based on mental illness severity; and (2) the treatment algorithm that should be followed to step up/down treatment intensity based on clinical response. In a recent Productivity Commission inquiry on mental health (Productivity Commission, 2020), service providers expressed a need for greater clarity on the operational aspects involved in implementing a stepped care model within their respective PHNs. The current study supports further investigation into one such model to treat depression/anxiety. Beyond this, there is emerging evidence on the effectiveness of digital platforms that can be readily implemented across GP clinics to initially match treatments for depression/anxiety on the basis of patient needs (Fletcher *et al*., 2021a, 2021b); though explicit step up/down algorithms need to be further developed and examined. Another study has evaluated the effectiveness/cost-effectiveness of an alternative stepped care model for the treatment of depression/anxiety in the Australian primary care setting (Anderson *et al*., 2019). However, the full evaluation is yet to be published.

Study findings should be interpreted with caution as they only indicate the potential cost-effectiveness of the CSC intervention under ‘steady-state’ conditions. Several factors can influence the real-world cost-effectiveness of the CSC intervention if implemented in practice. The first involves the establishment of well-defined treatment algorithms that are clearly communicated by healthcare providers and adhered to by patients, while allowing for flexibility around patient preferences. Second, is the need for systematic monitoring of patient outcomes using a simple, accurate screening tool that is accompanied by a coherent set of criteria to move patients up/down to a higher/lower intensity intervention. Third, a higher population density per mental health professional will improve the cost-effectiveness profile of the CSC intervention. Fourth, issues around the feasibility and acceptability of the CSC intervention may reduce its likelihood of being universally implemented across all Australian PHNs (e.g. not all GP clinics may wish to hire a full-time mental health nurse).

### Limitations

This study sourced best-available evidence to inform the cost-effectiveness model and underlying input parameters; and to apply conservative assumptions in the absence of data. Several limitations may impact on the validity of study findings. First, the cost-effectiveness model was based on an RCT conducted in the Netherlands (Oosterbaan *et al*., 2013). There was no Australian study examining the effectiveness of a comparable stepped care model for the treatment of depression/anxiety. It is unclear whether the health outcomes observed in the Dutch RCT are replicable in the Australian context. Second, the cost-effectiveness model may have underestimated health outcomes as it did not quantify treatment-related reductions in symptom severity that were not sufficient to meet remission criteria. Third, the exclusion of productivity impacts (absenteeism/presenteeism) from the analytic approach may underestimate potential economic benefits. Fourth, the underlying Dutch RCT (Oosterbaan *et al*., 2013) did not report outcomes for patients who had depression with psychotic features or were actively suicidal. The cost-effectiveness implications of including very severe cases of depression/anxiety are uncertain.

## Conclusion

This study found that a Dutch CSC intervention, with nurse-delivered guided self-help treatment as a first step, has the potential to be cost-effective in treating depression/anxiety if transferred to the Australian context. The CSC intervention would be a good candidate for adaptation and further testing as part of a broader stepped care approach implemented in the Australian primary care setting. However, further evidence is needed to verify the real-world cost-effectiveness of the CSC intervention when implemented in practice and to evaluate its effectiveness/cost-effectiveness when compared to other viable stepped care models for the treatment of depression/anxiety.

## Data Availability

Data supporting study findings are available from the corresponding author upon reasonable request.

## Supplementary material

For supplementary material accompanying this paper visit http://dx.doi.org/10.1017/S2045796021000470. click here to view supplementary material
