# Supplementary material for: Exploring the cost-effectiveness of a Dutch collaborative stepped care intervention for the treatment of depression and/or anxiety when adapted to the Australian context: a model-based cost-utility analysis
Source: Epidemiol Psychiatr Sci. 2021 Aug 25;30:e58. doi: 10.1017/S2045796021000470 (PMC8392705; doi:10.1017/S2045796021000470)
Supplement: Supplementary file 1 [file S2045796021000470sup001.pdf]

Online Supplementary Appendix

Table S1. Consolidated Health Economic Evaluation Reporting Standards (CHEERS) statement

Section/item	Item No	Recommendation	Section reported
**Title and abstract**			
Title	1	Identify the study as an economic evaluation or use more specific terms such as "cost-effectiveness analysis", and describe the interventions compared.	See Title
Abstract	2	Provide a structured summary of objectives, perspective, setting, methods (including study design and inputs), results (including base case and uncertainty analyses), and conclusions.	See Abstract
**Introduction**			
Background and objectives	3	Provide an explicit statement of the broader context for the study.	See Introduction
		Present the study question and its relevance for health policy or practice decisions.	See Introduction
**Methods**			
Target population and subgroups	4	Describe characteristics of the base case population and subgroups analysed, including why they were chosen.	See *Eligible population* subsection in the Methods
Setting and location	5	State relevant aspects of the system(s) in which the decision(s) need(s) to be made.	See Introduction and *Analytic approach* subsection in the Methods
Study perspective	6	Describe the perspective of the study and relate this to the costs being evaluated.	See *Analytic approach* subsection in the Methods
Comparators	7	Describe the interventions or strategies being compared and state why they were chosen.	See *Evidence of effectiveness* subsection in the Methods
Time horizon	8	State the time horizon(s) over which costs and consequences are being evaluated and say why appropriate.	See *Analytic approach* subsection in the Methods
Discount rate	9	Report the choice of discount rate(s) used for costs and outcomes and say why appropriate.	See *Analytic approach* subsection in the Methods
Choice of health outcomes	10	Describe what outcomes were used as the measure(s) of benefit in the evaluation and their relevance for the type of analysis performed.	See *Analytic approach* and *Health outcomes modelling* subsections in the Methods
Measurement of effectiveness	11a	*Single study-based estimates:* Describe fully the design features of the single effectiveness study and why the single study was a sufficient source of clinical effectiveness data.	See *Evidence of effectiveness* subsection in the Methods
	11b	*Synthesis-based estimates*: Describe fully the methods used for identification of included studies and synthesis of clinical effectiveness data.	Not applicable
Measurement and valuation of preference based outcomes	12	If applicable, describe the population and methods used to elicit preferences for outcomes.	Not applicable

Section/item	Item No	Recommendation	Section reported
Estimating resources and costs	13a	*Single study-based economic evaluation:* Describe approaches used to estimate resource use associated with the alternative interventions. Describe primary or secondary research methods for valuing each resource item in terms of its unit cost. Describe any adjustments made to approximate to opportunity costs.	Not applicable
	13b	*Model-based economic evaluation:* Describe approaches and data sources used to estimate resource use associated with model health states. Describe primary or secondary research methods for valuing each resource item in terms of its unit cost. Describe any adjustments made to approximate to opportunity costs.	See *Cost analysis* subsection in the Methods, Supplementary Text S6 and Supplementary Table S7
Currency, price date, and conversion	14	Report the dates of the estimated resource quantities and unit costs. Describe methods for adjusting estimated unit costs to the year of reported costs if necessary. Describe methods for converting costs into a common currency base and the exchange rate.	See Supplementary Table S7 alongside the *Analytic approach*, the *Cost analysis* subsections in the Methods and Supplementary Text S6
Choice of model	15	Describe and give reasons for the specific type of decision-analytical model used. Providing a figure to show model structure is strongly recommended.	See *Evidence of effectiveness* subsection in the Methods, alongside Figures 1 and 2
Assumptions	16	Describe all structural or other assumptions underpinning the decision-analytical model.	See Methods and Supplementary Text S2
Analytical methods	17	Describe all analytical methods supporting the evaluation. This could include methods for dealing with skewed, missing, or censored data; extrapolation methods; methods for pooling data; approaches to validate or make adjustments (such as half cycle corrections) to a model; and methods for handling population heterogeneity and uncertainty.	See Methods and Supplementary Materials S2 to S8
**Results**			
Study parameters	18	Report the values, ranges, references, and, if used, probability distributions for all parameters. Report reasons or sources for distributions used to represent uncertainty where appropriate. Providing a table to show the input values is strongly recommended.	See Table 1 and Supplementary Tables S4 and S7
Incremental costs and outcomes	19	For each intervention, report mean values for the main categories of estimated costs and outcomes of interest, as well as mean differences between the comparator groups. If applicable, report incremental cost-effectiveness ratios.	See Table 1 in the main manuscript and Supplementary Table S7
Characterising uncertainty	20a	*Single study-based economic evaluation:* Describe the effects of sampling uncertainty for the estimated incremental cost and incremental effectiveness parameters, together with the impact of methodological assumptions (such as discount rate, study perspective).	Not applicable
	20b	*Model-based economic evaluation:* Describe the effects on the results of uncertainty for all input parameters, and uncertainty related to the structure of the model and assumptions.	See Results, Table 1 and Supplementary Materials S4, S7 and S9-S12
Characterising heterogeneity	21	If applicable, report differences in costs, outcomes, or cost-effectiveness that can be explained by variations between subgroups of patients with different baseline characteristics or other observed variability in effects that are not reducible by more information.	Not applicable
**Discussion**			
Study findings, limitations, generalisability, and current knowledge	22	Summarise key study findings and describe how they support the conclusions reached. Discuss limitations and the generalisability of the findings and how the findings fit with current knowledge.	See Discussion

Section/item	Item No	Recommendation	Section reported
**Other**			
Source of funding	23	Describe how the study was funded and the role of the funder in the identification, design, conduct, and reporting of the analysis. Describe other non-monetary sources of support.	See Financial support
Conflicts of interest	24	Describe any potential for conflict of interest of study contributors in accordance with journal policy. In the absence of a journal policy, we recommend authors comply with International Committee of Medical Journal Editors recommendations.	See Conflicts of interests

Text S2. Description of excluded RCTs on the effectiveness of stepped care treatment for adult depression/anxiety

RCTs on the effectiveness of stepped care treatment for adult depression and/or anxiety were identified from: two previous systematic reviews [1,2]; and a search of PubMed and Google Scholar to find additional studies up to December 2020. In-scope RCTs for the cost-effectiveness model included those which: analysed stepped care treatment for both depression and/or anxiety among adults; involved a progressive increase in treatment intensity; contained sufficient data to inform cost-effectiveness modelling; universally targeted all patients with depression and/or anxiety; was generalizable to the Australian setting; and involved a CAU control arm reflective of services currently offered in Australia. A total of 12 RCTs examined stepped care models involving a progressive increase in treatment intensity. Of these, we excluded: four involving prevention [3-6]; one targeting elderly outpatients with diabetes [7]; one not generalizable to the Australian setting [8]; one involving treatment of anxiety only [9]; one involving treatment of depression only and with insufficient data to model changes in resource use [10]; one due to confounding of the treatment effect – i.e., the first step of the stepped care model (watchful waiting) was administered to both intervention and control arms [11]; one involving an active treatment in the control arm [12]; and one with insufficient data to inform cost-effectiveness modelling [13].

Table S3. Methods and data used to adjust prevalence estimates from the Global Burden of Disease Study 2019

Parameter	Value and uncertainty range	Uncertainty distribution	Source
Methods used to adjust GBD 2019 prevalence estimates			
First, GBD 2019 data on the prevalence of major depressive disorder, dysthymia and anxiety disorders were adjusted to only include individuals who were eligible to receive the stepped care intervention. This included those who: consulted with a GP for mental health problem in the past 12 months; did not have 12-month psychotic symptoms; did not have a 12-month bipolar disorder; and did not attempt suicide in the past 12 months. Second, the prevalence of depression was calculated by combining data on the prevalence of major depressive disorder and dysthymia, after accounting for comorbidity between the two disorders. Third, the prevalence of anxiety was adjusted to exclude the prevalence of obsessive-compulsive disorder and post-traumatic stress disorder, which were both included in the GBD 2019 prevalence estimates. Fourth, the GBD 2019 prevalence estimates did not account for comorbidity between depression and anxiety. A correction was made by calculating the overlap between the prevalence of depression and anxiety; and assigned all co-morbid cases to a single disorder based on the proportion who reported depression or anxiety as the primary disorder.			
Data used to adjust GBD 2019 prevalence estimates			
Unadjusted prevalence of major depressive disorder (depression)	15-19 years: M - 3.3% (0.6); F - 5.9% (1.1)<br>20-24 years: M - 4.3% (0.8); F - 7.7% (1.5)<br>25-29 years: M - 3.9% (0.7); F - 6.7% (1.2)<br>30-34 years: M - 3.8% (0.8); F - 5.9% (1.2)<br>35-39 years: M - 4.1% (0.6); F - 5.9% (0.8)<br>40-44 years: M - 4.2% (0.7); F - 5.7% (0.9)<br>45-49 years: M - 3.9% (0.6); F - 5.2% (0.7)<br>50-54 years: M - 3.6% (0.5); F - 4.6% (0.6)<br>55-59 years: M - 3.2% (0.5); F - 4.1% (0.6)<br>60-64 years: M - 3.0% (0.5); F - 3.7% (0.7)<br>65-69 years: M - 2.9% (0.4); F - 3.5% (0.5)<br>70-74 years: M - 2.8% (0.4); F - 3.3% (0.4)<br>75-79 years: M - 2.7% (0.5); F - 3.1% (0.6)<br>80-84 years: M - 2.7% (0.5); F - 3.1% (0.6)<br>85-89 years: M - 2.7% (0.4); F - 3.3% (0.5)<br>90-94 years: M - 2.7% (0.5); F - 3.5% (0.7)<br>95+ years: M - 2.7% (0.6); F - 3.8% (0.9)	Beta	[14]
Unadjusted prevalence of dysthymia (depression)	15-19 years: M - 0.6% (0.1); F - 1.0% (0.3)<br>20-24 years: M - 1.0% (0.2); F - 1.6% (0.4)<br>25-29 years: M - 1.2% (0.3); F - 1.9% (0.4)<br>30-34 years: M - 1.3% (0.3); F - 2.0% (0.4)<br>35-39 years: M - 1.4% (0.3); F - 2.0% (0.4)<br>40-44 years: M - 1.4% (0.3); F - 2.0% (0.4)<br>45-49 years: M - 1.4% (0.3); F - 1.9% (0.4)<br>50-54 years: M - 1.3% (0.3); F - 1.8% (0.4)<br>55-59 years: M - 1.2% (0.2); F - 1.7% (0.3)<br>60-64 years: M - 1.2% (0.3); F - 1.5% (0.3)<br>65-69 years: M - 1.1% (0.2); F - 1.4% (0.3)<br>70-74 years: M - 1.0% (0.2); F - 1.3% (0.2)<br>75-79 years: M - 1.0% (0.2); F - 1.1% (0.2)<br>80-84 years: M - 1.0% (0.2); F - 1.0% (0.2)<br>85-89 years: M - 1.0% (0.2); F - 1.0% (0.2)<br>90-94 years: M - 1.0% (0.2); F - 1.0% (0.2)<br>95+ years: M - 1.0% (0.3); F - 1.0% (0.3)	Beta	[14]

Parameter	Value and uncertainty range	Uncertainty distribution	Source
Unadjusted prevalence of anxiety disorders (anxiety)	15-19 years: M - 6.1% (1.0); F - 8.4% (1.4)<br>20-24 years: M - 6.4% (1.3); F - 9.9% (2.0)<br>25-29 years: M - 6.4% (1.4); F - 10.5% (2.2)<br>30-34 years: M - 6.3% (1.2); F - 10.6% (2.0)<br>35-39 years: M - 6.2% (1.1); F - 10.4% (1.8)<br>40-44 years: M - 6.0% (1.2); F - 10.0% (1.8)<br>45-49 years: M - 5.6% (1.2); F - 9.4% (1.9)<br>50-54 years: M - 5.1% (0.9); F - 8.5% (1.6)<br>55-59 years: M - 4.4% (0.8); F - 7.5% (1.3)<br>60-64 years: M - 3.9% (0.8); F - 6.5% (1.2)<br>65-69 years: M - 3.4% (0.7); F - 5.8% (1.2)<br>70-74 years: M - 3.1% (0.6); F - 5.2% (1.1)<br>75-79 years: M - 2.8% (0.5); F - 4.7% (0.9)<br>80-84 years: M - 2.5% (0.5); F - 4.3% (0.8)<br>85-89 years: M - 2.1% (0.4); F - 3.8% (0.7)<br>90-94 years: M - 1.8% (0.4); F - 3.3% (0.7)<br>95+ years: M - 1.4% (0.3); F - 2.8% (0.6)	Beta	[14]
Proportion of those with a common mental disorder who are eligible to receive the stepped care intervention [a]	Males<br>Major depression: 26.0% (SE: 9.6)<br>Dysthymia: 29.5% (SE: 10.1)<br>Anxiety disorders: 19.5% (SE: 4.4)<br>Females<br>Major depression: 43.7% (SE: 7.9)<br>Dysthymia: 42.6% (SE: 15.8)<br>Anxiety disorders: 26.0% (SE: 2.4)	Beta	[15]
Relative split between major depression and dysthymia among those with a depressive disorder	Major depression: 67.1% (SE: 6.8)<br>Dysthymia: 5.7% (SE: 2.6)<br>Both disorders: 27.2% (SE: 7.0)	Dirichlet	[15]
Proportion who have an in-scope anxiety disorder among those with any anxiety disorder [b]	In-scope disorder: 58.6% (SE: 6.8)<br>Out-of-scope disorder: 41.4% (SE: 6.8)	Beta	[15]
Relative split between depressive disorders and anxiety disorders	Males<br>Depressive disorders: 42.2% (SE: 13.1)<br>Anxiety disorders: 46.7% (SE: 11.6)<br>Both disorders: 11.0% (SE: 4.9)<br>Females<br>Depressive disorders: 28.9% (SE: 5.9)<br>Anxiety disorders: 46.8% (SE: 5.8)<br>Both disorders: 24.3% (SE: 4.9)	Dirichlet	[15]
Proportion who consider depression or anxiety as the main problem among those with a comorbid depressive/anxiety disorder	The main problem involves:<br>Depressive disorders: 45.5% (SE: 11.8)<br>Anxiety disorders: 54.5% (SE: 11.8)	Beta	[15]

Abbreviations: F - female; GBD - Global Burden of Disease; M - male; N/A - not applicable; SE - standard error.

[a] *This includes everyone in the 2007 National Survey of Mental Health and Wellbeing who: visited a GP in the past year; did not have 12-month psychosis; did not attempt suicide in the past year; and did not have a 12-month bipolar disorder.*

[b] *In-scope anxiety disorders include panic disorder, agoraphobia, social phobia and generalised anxiety disorder. Out-of-scope disorders include: obsessive-compulsive disorder and post-traumatic stress disorder.*

Table S4. Input parameters and uncertainty ranges for health outcome modelling

Parameter	Value and uncertainty range	Uncertainty distribution	Source
2019 Australian population	18-24 years: M - 1,223,509; F - 1,155,990<br>25-34 years: M - 1,891,333; F - 1,908,521<br>35-44 years: M - 1,679,072; F - 1,699,537<br>45-54 years: M - 1,576,468; F - 1,639,088<br>55-64 years: M - 1,435,273; F - 1,504,814<br>65-74 years: M - 1,115,385; F - 1,170,310<br>75-84 years: M - 579,015; F - 660,450<br>85+ years: M - 197,062; F - 317,908<br>Total: M - 9,697,117; F - 10,056,618	N/A	[16]
Prevalence of depression	18-24 years: M - 1.0% (SE: 0.4); F - 3.1% (SE: 0.8)<br>25-34 years: M - 1.0% (SE: 0.4); F - 2.6% (SE: 0.7)<br>35-44 years: M - 1.1% (SE: 0.4); F - 2.4% (SE: 0.6)<br>45-54 years: M - 1.0% (SE: 0.4); F - 2.1% (SE: 0.5)<br>55-64 years: M - 0.8% (SE: 0.3); F - 1.6% (SE: 0.4)<br>65-74 years: M - 0.8% (SE: 0.3); F - 1.4% (SE: 0.3)<br>75-84 years: M - 0.7% (SE: 0.3); F - 1.3% (SE: 0.3)<br>85+ years: M - 0.7% (SE: 0.3); F - 1.5% (SE: 0.4)<br>Total: M - 0.9% (SE: 0.4); F - 2.1% (SE: 0.4)	Beta	[14,15]
Prevalence of anxiety	18-24 years: M - 0.7% (SE: 0.2); F - 1.2% (SE: 0.3)<br>25-34 years: M - 0.7% (SE: 0.2); F - 1.4% (SE: 0.3)<br>35-44 years: M - 0.6% (SE: 0.2); F - 1.3% (SE: 0.3)<br>45-54 years: M - 0.6% (SE: 0.2); F - 1.2% (SE: 0.3)<br>55-64 years: M - 0.4% (SE: 0.1); F - 0.9% (SE: 0.2)<br>65-74 years: M - 0.3% (SE: 0.1); F - 0.7% (SE: 0.2)<br>75-84 years: M - 0.3% (SE: 0.1); F - 0.6% (SE: 0.1)<br>85+ years: M - 0.2% (SE: 0.1); F - 0.4% (SE: 0.1)<br>Total: M - 0.5% (SE: 0.1); F - 1.1% (SE: 0.2)	Beta	[14,15]
Severity distribution for depression and anxiety	Depression<br>Mild: 79.5% (SE: 3.6)<br>Moderate: 12.4% (SE: 1.3)<br>Severe: 8.0% (SE: 2.6)<br>Anxiety<br>Mild: 57.0% (SE: 5.0)<br>Moderate: 25.8% (SE: 3.6)<br>Severe: 17.2% (SE: 3.2)	Dirichlet	[14]
Disability weights for remitted and unremitted health states	Depression (unremitted): 0.401 (SE: 0.027) [a]<br>Anxiety (unremitted): 0.267 (SE: 0.018) [a]<br>No disorder (remitted): 0.146 (SE: 0.005) [b]	Beta	[14]
Remission outcomes in the CSC intervention presented as 4-month probabilities<br>(by disorder and follow-up)	Depression<br>4 months: 48.9% (SE: 7.2)<br>8 months: 55.3% (SE: 7.2)<br>12 months: 63.8% (SE: 6.9)<br>Anxiety<br>4 months: 55.2% (SE: 9.1)<br>8 months: 55.2% (SE: 9.1)<br>12 months: 75.9% (SE: 7.8)	Beta	[17]

Parameter	Value and uncertainty range	Uncertainty distribution	Source
Remission outcomes in the CAU comparator presented as 4-month probabilities<br><br>(by disorder and follow-up)	Depression<br>4 months: 23.1% (SE: 8.1)<br>8 months: 38.5% (SE: 9.4)<br>12 months: 42.3% (SE: 9.5)<br>Anxiety<br>4 months: 25.9% (SE: 8.1)<br>8 months: 48.1% (SE: 9.3)<br>12 months: 66.7% (SE: 8.8)	Beta	[17]

Abbreviations: CAU - care-as-usual; CSC - collaborative stepped care; F - female; M - male; N/A - not applicable; SE - standard error.

[a] *This disorder-specific disability weight is inclusive of background morbidity due to other causes of disease and injury beyond depression/anxiety (see Supplementary Text S2).*

[b] *This disability weight represents background morbidity due to other causes of disease and injury among individuals who do not have depression/anxiety (see Supplementary Text S2).*

Text S5. Methods used to estimate disability weights for depression, anxiety and background morbidity

Disability weights for remitted and unremitted cases of depression/anxiety were estimated as follows. Disability weights were obtained by severity level for depression and anxiety from the Global Burden of Disease Study 2019 (GBD 2019) [14]. Data on the proportion of mild, moderate and severe cases (i.e., the severity distribution) were also sourced from GBD 2019 [14]. Both sets of data are presented in the table below. The weighted-average disability weight was estimated for each disorder by using the severity distribution to comparatively weight each severity-specific disability weight. This resulted in a weighted-average disability weight of 0.298 (SE: 0.032) for depression and 0.141 (SE: 0.021) for anxiety. These disorder-specific disability weights do not, however, account for background morbidity due to other causes of disease apart from depression/anxiety.

Disorder severity	Severity distribution	Disability weight
Depression		
Mild	79.5% (SE: 3.6)	0.145 (SE: 0.028)
Moderate	12.4% (SE: 1.3)	0.396 (SE: 0.067)
Severe	8.0% (SE: 2.6)	0.658 (SE: 0.084)
Total	*100.0%*	*0.298 (SE: 0.032)*
Anxiety		
Mild	57.0% (SE: 5.0)	0.030 (SE: 0.007)
Moderate	25.8% (SE: 3.6)	0.133 (SE: 0.024)
Severe	17.2% (SE: 3.2)	0.523 (SE: 0.080)
Total	*100.0%*	*0.141 (SE: 0.021)*

To incorporate background morbidity into the disorder-specific disability weights, GBD 2019 data was first obtained on the rate of prevalent years lived with disability (PYLD) for each disorder. The table below presents 2019 Australian data on the PYLD rate (by sex and age group) attributable to: all causes of disease and injury (inclusive of depression/anxiety); anxiety only; and depression only. The PYLD rate for other causes of disease and injury (excluding depression/anxiety) was estimated by subtracting the sum of PYLD rates for depression and anxiety from the all-cause PYLD rate. Data on the 2019 Australian population (see Table 1 in the manuscript) [16] was used to estimate the weighted-average PYLD rate due to other causes of disease and injury (across all sexes and age groups). This was estimated to be 0.146 (SE: 0.005) and became the disability weight for remitted cases of depression/anxiety.

Sex and age group	PYLD rate (per 1) – All causes	PYLD rate (per 1) – Anxiety	PYLD rate (per 1) – Depression [a]	PYLD rate (per 1) – Other causes
Male				
18-24 years	0.094 (SE: 0.013)	0.006 (SE: 0.001)	0.010 (SE: 0.002)	0.078 (SE: 0.013)
25-34 years	0.111 (SE: 0.013)	0.006 (SE: 0.001)	0.009 (SE: 0.002)	0.096 (SE: 0.013)
35-44 years	0.124 (SE: 0.014)	0.006 (SE: 0.001)	0.010 (SE: 0.002)	0.108 (SE: 0.014)
45-54 years	0.137 (SE: 0.016)	0.005 (SE: 0.001)	0.009 (SE: 0.001)	0.123 (SE: 0.016)
55-64 years	0.170 (SE: 0.020)	0.004 (SE: 0.001)	0.007 (SE: 0.001)	0.159 (SE: 0.020)
65-74 years	0.228 (SE: 0.024)	0.003 (SE: 0.001)	0.006 (SE: 0.001)	0.218 (SE: 0.024)
75-84 years	0.290 (SE: 0.034)	0.002 (SE: 0.001)	0.006 (SE: 0.001)	0.282 (SE: 0.034)
85+ years	0.372 (SE: 0.046)	0.001 (SE: 0.000)	0.006 (SE: 0.001)	0.365 (SE: 0.046)
Female				
18-24 years	0.116 (SE: 0.016)	0.009 (SE: 0.002)	0.015 (SE: 0.004)	0.089 (SE: 0.017)
25-34 years	0.137 (SE: 0.016)	0.010 (SE: 0.002)	0.013 (SE: 0.003)	0.112 (SE: 0.016)
35-44 years	0.149 (SE: 0.017)	0.010 (SE: 0.002)	0.012 (SE: 0.002)	0.126 (SE: 0.018)
45-54 years	0.161 (SE: 0.019)	0.008 (SE: 0.002)	0.010 (SE: 0.002)	0.141 (SE: 0.019)
55-64 years	0.183 (SE: 0.021)	0.006 (SE: 0.001)	0.008 (SE: 0.001)	0.168 (SE: 0.021)
65-74 years	0.228 (SE: 0.025)	0.005 (SE: 0.001)	0.006 (SE: 0.001)	0.216 (SE: 0.025)
75-84 years	0.294 (SE: 0.034)	0.004 (SE: 0.001)	0.006 (SE: 0.001)	0.283 (SE: 0.034)
85+ years	0.404 (SE: 0.048)	0.003 (SE: 0.001)	0.006 (SE: 0.002)	0.395 (SE: 0.048)

[a] The PYLD rate for depression is the sum of PYLD rates for major depressive disorder and dysthymia which are both considered depressive disorders in GBD 2019

Raw disability weights for depression/anxiety (excluding background morbidity) are presented in the table below, alongside the disability weight for other causes of disease and injury (i.e., background morbidity). A multiplicative function, commonly used in Global Burden of Disease studies and described elsewhere [18], was employed to estimate the disability weight for each disorder inclusive of background morbidity.

Cause of disease	Raw disability weight	Disability weight inclusive of background morbidity
Depression (unremitted)	0.298 (SE: 0.032)	0.401 (SE: 0.027)
Anxiety (unremitted)	0.141 (SE: 0.021)	0.267 (SE: 0.018)
Other causes (remitted)	0.146 (SE: 0.005)	0.146 (SE: 0.005)

Text S6. Methods and data sources for the cost analysis

Overview

Intervention pathways for the CSC intervention and the CAU comparator are presented in Figure 1 and Figure 2 of the main manuscript, respectively. These pathways were used to estimate costs for the CSC intervention and CAU comparator. The flow of patients through each node in the intervention pathway was based on data from Oosterbaan et al. [17]. Supplementary 0 presents input parameters used to enumerate costs in the CSC intervention and CAU comparator. Costs for the CSC intervention were broadly grouped into four categories: the cost of training healthcare providers (CSC 0); the first step for mild disorders involving guided self-help only (CSC 1a); the first step for moderate disorders comprising guided self-help plus antidepressant medication (CSC 1b); and the second step involving specialised mental healthcare (CSC 2a, 2b & 2c). Each step in the CSC intervention comprised several line items that were costed as the product of: the total number of people to which the cost applies; the duration of the intervention step (measured in months); and the unit cost per person-month of the specified item. For example, the duration of the first step was 4 months (CSC 1a & 1b), while the duration of the second step was 4 months for those who did not remit during the first step (CSC 2b & 2c) and 8 months for those with a severe disorder at baseline (CSC 2a).

CSC intervention – Training healthcare providers

Training of healthcare providers (CSC 0) encompassed: (1) initial training of GPs and GP mental health nurses at baseline; and (2) ongoing training of GPs and GP mental health nurses throughout the 8-month intervention delivery period.

At baseline, GPs received one educational session from a psychiatrist to clarify the medication algorithm and disseminate advice on promoting medication adherence. The educational session was assumed to be delivered in a group format with an average attendance of 20 persons per session and an average duration of one hour. The total number of groups that would receive an educational session (1,555 groups) was estimated by: taking the total number of GPs in Australia [19]; and dividing this by the average group size of 20 persons per group. The total time spent training GPs at baseline was the product of the total number of groups, the number of educational sessions per group and the duration of each educational session. Ongoing training was provided by a psychiatrist to each individual GP throughout the 8-month intervention delivery period. This provided an opportunity for direct, case-based consultation with the psychiatrist and occurred about once every two months.

At baseline, GP mental health nurses participated in a 2-day group training session facilitated by a trained psychologist to educate them on the provision of guided self-help and basic cognitive behavioural therapy (CBT) strategies. The number of GP mental health nurses who underwent training (5,464 nurses) was estimated by: calculating the total number of GP clinics in Australia – i.e., the total number of GPs in Australia [19] divided by the average number of GPs per clinic [20]; then assuming that there was one GP mental health nurse per GP clinic. The total number of group training sessions (273 sessions) was then estimated by dividing the total number of GP mental health nurses in Australia

by the average group size of 20 persons per group. The total time spent training GP mental health nurses (4,098 hours) was then estimated as the product of: the total number of 2-day group training sessions; and the total duration of a 2-day group training session (15 hours).

Hourly wage rates for psychiatrists and psychologists who provide training were taken from the ABS Survey of Employee Earnings and Hours [21], plus a 30% loading to incorporate on-costs – i.e., wage loadings to account for administration costs, personal leave, superannuation, etc.

CSC intervention – Guided self-help

Guided self-help for people with a mild-to-moderate depression/anxiety (CSC 1a) involved patients participating in a 4-month self-help course comprising five 45-minute sessions, with guidance from a GP mental health nurse at a primary care clinic or at home. Patients with depression participated in the 'Coping with Depression' course [22], while those with anxiety participated in the 'Stresspac' course [23]. Both courses consisted of workbooks with psychoeducation and CBT exercises. This was applied to the Australian context by assuming that all GP mental health nurses and eligible patients had their own copy of the relevant self-help book costed at the recommended retail price (range of prices obtained via Google search).

CSC intervention – Medication algorithms

In addition to receiving guided self-help, patients with a moderate depression/anxiety (CSC 1b) were offered antidepressant medication according to disorder-specific medication algorithms. All patients with depression/anxiety were initially prescribed a selective serotonin reuptake inhibitor (SSRI). In the case of non-response or low tolerability, patients were switched to: a serotonin-norepinephrine reuptake inhibitor (SNRI), tricyclic antidepressant (TCA) or tetracyclic antidepressant (TeCA) if they had depression; an SNRI or other SSRI if they had generalised anxiety disorder; or another SSRI if they had panic disorder with/or without agoraphobia. In the model, the probability of switching medications following first-line treatment with an SSRI was based on data from the Sequenced Treatment Alternatives to Relieve Depression (STAR*D) study [24]. The timing of the switch was modelled using a uniform distribution – i.e., assuming the switch had an equal probability of occurring across all time points during the intervention step. The monthly unit price for each drug class was estimated using data from the Pharmaceutical Benefits Scheme (PBS) [25].

CSC intervention – Specialised mental healthcare

Patients receiving specialised mental healthcare (CSC 2a, 2b & 2c) could obtain treatment using antidepressants, psychotherapy or both. However, the study did not describe the number of patients allocated to each treatment or the circumstances involved. Unpublished data were obtained from the Diagnosis, Management and Outcomes of Depression in the Primary Care Setting (*diamond*) study to estimate the proportion receiving each type of treatment in specialised mental healthcare [26]. Data on the proportion of patients (with moderate-to-severe depressive symptoms) who saw a GP only, a GP

plus a psychologist or a GP plus a psychiatrist were used as a proxy for the proportion of patients who received antidepressants only, psychotherapy only or a combination of both, respectively. Patients who saw a GP, psychologist and psychiatrist were included among the group who saw a GP plus a psychiatrist. It was assumed that medication algorithms for patients in the second step were identical to those applied to patients in the first step. Furthermore, patients who only received psychotherapy were assumed to see a clinical psychologist 8 times (range: 6-10); while patients who received psychotherapy plus antidepressants were assumed to see a psychiatrist 8 times (range: 6-10). The point estimate for the number of psychologist/psychiatrist visits was based on commonly used thresholds for minimally adequate treatment [27], while the range was based on current Medicare guidelines which subsidise a maximum of 10 psychotherapy sessions each year [28].

CAU comparator

The types of treatments received by patients in the CAU comparator, included: treatment with antidepressants only (CAU 1); referral to specialised mental healthcare (CAU 2); and receiving no treatment (CAU 3). Patients who received antidepressant medication were costed using the same medication algorithms as those outlined in the CSC intervention. Likewise, patients receiving specialised mental healthcare were costed in a similar manner to those in the CSC intervention. Patients who dropped out were included among those who received no treatment. All patients who received no treatment were assumed to incur zero costs, which is a conservative assumption given that additional costs in the CAU comparator will lead to a lower (i.e., more cost-effective) ICER. The duration of each treatment in the CAU comparator was 8 months.

Benzodiazepine use

GPs were free to prescribe benzodiazepines to all patients on top of their existing treatments in the CSC intervention and CAU comparator over the 8-month study period (CSC 9 and CAU 9, respectively). The cost of benzodiazepine use was calculated in a similar manner to the medication algorithms outlined previously, but based on the proportion who reported using benzodiazepines in each group.

Unit cost of health professional visits (CSC intervention and CAU comparator)

All medical consultations with a GP, psychologist or psychiatrist were costed using weighted average of all relevant fees from the Medicare Benefits Schedule (MBS) [28]. In Australia, commencement of mental health treatment in the formal mental healthcare system always begins with an initial GP consult to develop a GP mental health care plan. It was assumed that all patients who begin mental health treatment at 0 months (baseline) had an initial visit with a GP to develop a GP mental health care plan (MBS item no: 2700 to 2717). Consultations with a GP mental health nurse were costed by using the weighted average fee for MBS items involving focussed psychological strategies with an allied health professional (i.e., registered psychologist, occupational therapist or social worker) as a proxy for a similar service provided by a nurse (MBS item no: 80100 to 80165). The unit cost for other health professional visits included: GP visits to obtain medication repeats (MBS item no: 23); psychologist visits to undergo psychotherapy (MBS item no: 80000 to 80115); and psychiatrist visits to undergo psychotherapy (MBS item no: 304 to 308).

Cost offsets

Cost offsets were calculated by estimating the difference in treatment costs for unremitted cases of depression/anxiety between the CSC intervention and the relevant comparator. The average treatment cost for an unremitted case of depression/anxiety was based on data from a recent cost of illness study analysing the 2007 National Survey of Mental Health and Wellbeing [29].

Table S7. Input parameters and uncertainty ranges for the cost analysis

Parameter	Value and uncertainty range (when applicable)	Uncertainty distribution	Sources
	Treatment allocation among patients in the CSC intervention and CAU comparator		
Treatments received by patients in the CSC intervention	Of those with a mild-to-moderate disorder (n=88):<br>33.0% go to CSC 1a<br>56.8% go to CSC 1b<br>10.2% drop out and do not start self-help<br>Of those with a severe disorder (n=6):<br>100.0% go to CSC 2a<br>Of those who go to CSC 1a & 1b (n=79):<br>55.7% achieve full remission<br>25.3% do not remit & go to CSC 2b<br>19.0% decline further treatment<br>Of those who achieve full remission (n=44):<br>84.1% stay remitted<br>15.9% relapse & go to CSC 2c	Dirichlet	RCT [17]
Treatments received by patients in the CAU comparator	Of those allocated to CAU (n=64):<br>35.9% were prescribed antidepressants (CAU 1)<br>43.8% received specialised mental healthcare (CAU 2)<br>20.3% dropped out (CAU 3)	Dirichlet	RCT [17]
	CSC intervention: Training of healthcare providers (CSC 0)		
Group training of GPs at baseline to deliver medication algorithms in the CSC intervention (0 months)	Wage rate for a psychiatrist: $119.29 per hour (SE: 19.76) [a]	Lognormal	[21]
	Time spent training GPs at baseline: 1,555 hours	-	Calculation
	Duration of each educational session: 1 hour<br>*No. of educational sessions per group: 1 session*<br>*No. of groups: 1,555 groups*<br>*Group size: 20 persons per group (range: 10 - 30)*<br>*No. of GPs in Australia: 31,102 persons*	-<br>-<br>Pert<br>-<br>-	Assumption<br>[17]<br>Calculation<br>Assumption<br>[19]
	A psychiatrist delivered 1 educational session to GPs in groups comprising 20 persons per group. Each educational session had an average duration of 1 hour.		
Ongoing individualised training of GPs over the course of the CSC intervention (0-8 months)	Wage rate for a psychiatrist: $119.29 per hour (SE: 19.76) [a]	Lognormal	[21]
	Time spent providing ongoing training GPs: 124,408 hours	-	Calculation
	Duration of each individualised session: 1 hour<br>*No. of sessions per GP over 8-month period: 4 sessions*<br>*No. of GPs in Australia: 31,102 persons*	-<br>-<br>-	Assumption<br>[17]<br>[19]
	Across the 8-month intervention delivery period, each GP received one individualised training session every two months from a psychiatrist.		

Parameter	Value and uncertainty range (when applicable)	Uncertainty distribution	Sources
Training of GP mental health nurses at baseline to deliver guided self-help in the CSC intervention (0 months)	Wage rate for a psychologist: $49.61 per hour (SE: 4.15) [a]	Lognormal	[21]
	Time spent training GP MH nurses at baseline: 4,098 hours	-	Calculation
	Duration of 2-day group training session: 15 hours	-	[17]
	No. of 2-day group training sessions: 273 sessions	-	Calculation
	Group size: 20 persons per group (range: 10 - 30)	Pert	Assumption
	No. of GP MH nurses in Australia: 5,464 persons	-	Calculation
	No. of GP MH nurses per GP clinic: 1 person per clinic	-	Assumption
	No. of GP clinics in Australia: 5,464 clinics	-	Calculation
	No. of GPs per GP clinic: 5.692 persons per clinic	-	[20]
	No. of GPs in Australia: 31,102 persons	-	[19]
	Self-help book (depression): $36.77 (range: 28.55 - 44.99)	Pert	Google search
	Self-help book (anxiety): $24.59 (range: 19.92 - 29.25)	Pert	Google search
	GP MH nurses participated in a 2-day training session in basic CBT strategies at baseline. Training sessions comprised 20 nurses per group. Each GP MH nurse received a copy of the self-help book for depression/anxiety.		
Ongoing group-based training of GP mental health nurses over the course of the CSC intervention (0-8 months)	Wage rate for a psychologist: $49.61 per hour (SE: 4.15) [a]	Lognormal	[21]
	Time spent supervising GP MH nurses: 4,735 hours	-	Calculation
	Duration of each group supervision session: 1 hour	-	Assumption
	No. of sessions per group: 17 sessions per group	-	[17]
	No. of groups undergoing supervision: 273 groups	-	Calculation
	Group size: 20 persons per group (range: 10 - 30)	Pert	Assumption
	No. of GP MH nurses in Australia: 5,464 persons	-	Calculation
	No. of GP MH nurses per GP clinic: 1 person per clinic	-	Assumption
	No. of GP clinics in Australia: 5,464 clinics	-	Calculation
	No. of GPs per GP clinic: 5.69 persons per clinic	-	[20]
	No. of GPs in Australia: 31,102 persons	-	[19]
	GP MH nurses undertook group supervision to monitor feedback and adherence to the self-help manual for depression/anxiety. This occurred once every 2 weeks with an experienced psychologist in groups comprising 20 nurses per group.		
	CSC intervention: Guided self-help only (CSC 1a)		
Visit to prepare GP mental health treatment plan (0 months) [b]	Cost of an initial GP visit: $98.67	-	[28]
Guided self-help with a GP mental health nurse (0-4 months)	Self-help book (depression): $36.77 (range: 28.55 - 44.99)	Pert	Google search
	Self-help book (anxiety): $24.59 (range: 19.92 - 29.25)	Pert	Google search
	No. of guided self-help sessions per patient: 5 sessions	-	[17]
	Cost of nurse per guided self-help session: $88.02	-	[28]
	CSC intervention: Guided self-help plus antidepressants (CSC 1b)		
Visit to prepare GP mental health treatment plan (0 months) [b]	Cost of an initial GP visit: $98.67	-	[28]
Guided self-help with a GP mental health nurse (0-4 months)	Self-help book (depression): $36.77 (range: 28.55 - 44.99)	Pert	Google search
	Self-help book (anxiety): $24.59 (range: 19.92 - 29.25)	Pert	Google search
	No. of guided self-help sessions per patient: 5 sessions	-	[17]
	Cost of nurse per guided self-help session: $88.02	-	[28]

Parameter	Value and uncertainty range (when applicable)	Uncertainty distribution	Sources
Medication algorithm for depression (0-4 months)	Proportion switching to 2nd-line treatment: 51.6% (SE: 0.8)<br>Cost of 1st-line treatment: $9.91 per month<br>Cost of 2nd-line treatment (post-switch): $17.74 per month<br>GP visits for medication repeats: $37.85 per month	Beta<br>-<br>-<br>-	[24]<br>[25]<br>[25]<br>[28]
Medication algorithm for anxiety (0-4 months)	Proportion switching to 2nd-line treatment: 51.6% (SE: 0.8)<br>Cost of 1st-line treatment: $9.91 per month<br>Cost of 2nd-line treatment (post-switch): $10.38 per month<br>GP visits for medication repeats: $37.85 per month	Beta<br>-<br>-<br>-	[24]<br>[25]<br>[25]<br>[28]
	CSC intervention: Specialised mental healthcare (CSC 2a)		
Visit to prepare GP mental health treatment plan (0 months) [b]	Cost of an initial GP visit: $98.67	-	[28]
Allocation of patients to different treatments in specialised mental healthcare (0-8 months)	Medication only: 51.0% (N: 158)<br>Psychotherapy only: 25.8% (N: 80)<br>Combination of both: 23.2% (N: 72)	Dirichlet	[26]
Medication algorithm for depression (0-8 months)	Proportion switching to 2nd-line treatment: 51.6% (SE: 0.8)<br>Cost of 1st-line treatment: $9.91 per month<br>Cost of 2nd-line treatment (post-switch): $17.74 per month<br>GP visits for medication repeats: $37.85 per month	Beta<br>-<br>-<br>-	[24]<br>[25]<br>[25]<br>[28]
Medication algorithm for anxiety (0-8 months)	Proportion switching to 2nd-line treatment: 51.6% (SE: 0.8)<br>Cost of 1st-line treatment: $9.91 per month<br>Cost of 2nd-line treatment (post-switch): $10.38 per month<br>GP visits for medication repeats: $37.85 per month	Beta<br>-<br>-<br>-	[24]<br>[25]<br>[25]<br>[28]
Psychologist visits among patients who receive psychotherapy only (0-8 months)	No. of psychotherapy sessions: 6 (range: 8 - 10)<br>Cost per psychologist session: $106.75	Pert<br>-	Assumption<br>[28]
Psychiatrist visits among patients who receive combination therapy (0-8 months)	No. of psychotherapy sessions: 6 (range: 8 - 10)<br>Cost per psychiatrist session: $164.39	Pert<br>-	Assumption<br>[28]
	CSC intervention: Specialised mental healthcare (CSC 2b & 2c)		
Allocation of patients to different treatments in specialised mental healthcare (4-8 months)	Medication only: 51.0% (N: 158)<br>Psychotherapy only: 25.8% (N: 80)<br>Combination of both: 23.2% (N: 72)	Dirichlet	[26]
Medication algorithm for depression (4-8 months)	Proportion switching to 2nd-line treatment: 51.6% (SE: 0.8)<br>Cost of 1st-line treatment: $9.91 per month<br>Cost of 2nd-line treatment (post-switch): $17.74 per month<br>GP visits for medication repeats: $37.85 per month	Beta<br>-<br>-<br>-	[24]<br>[25]<br>[25]<br>[28]
Medication algorithm for anxiety (4-8 months)	Proportion switching to 2nd-line treatment: 51.6% (SE: 0.8)<br>Cost of 1st-line treatment: $9.91 per month<br>Cost of 2nd-line treatment (post-switch): $10.38 per month<br>GP visits for medication repeats: $37.85 per month	Beta<br>-<br>-<br>-	[24]<br>[25]<br>[25]<br>[28]
Psychologist visits among patients who receive psychotherapy only (4-8 months)	No. of psychotherapy sessions: 6 (range: 8 - 10)<br>Cost per psychologist session: $106.75	Pert<br>-	Assumption<br>[28]

Parameter	Value and uncertainty range (when applicable)	Uncertainty distribution	Sources
Psychiatrist visits among patients who receive combination therapy (4-8 months)	No. of psychotherapy sessions: 6 (range: 8 - 10)<br>Cost per psychiatrist session: $164.39	Pert<br>-	Assumption<br>[28]
	CSC intervention: Benzodiazepine use (CSC 9)		
All patients who are prescribed benzodiazepines (0-8 months)	8-month probability of benzodiazepine use: 17.7% (SE: 3.9)<br>Cost of benzodiazepines: $14.58 per month	Beta<br>-	[17]<br>[25]
	CAU comparator: Prescribed antidepressants (CAU 1)		
Visit to prepare GP mental health treatment plan (0 months) [b]	Cost of an initial GP visit: $98.67	-	[28]
Medication algorithm for depression (0-8 months)	Proportion switching to 2nd-line treatment: 51.6% (SE: 0.8)<br>Cost of 1st-line treatment: $9.91 per month<br>Cost of 2nd-line treatment (post-switch): $17.74 per month<br>GP visits for medication repeats: $37.85 per month	Beta<br>-<br>-<br>-	[24]<br>[25]<br>[25]<br>[28]
Medication algorithm for anxiety (0-8 months)	Proportion switching to 2nd-line treatment: 51.6% (SE: 0.8)<br>Cost of 1st-line treatment: $9.91 per month<br>Cost of 2nd-line treatment (post-switch): $10.38 per month<br>GP visits for medication repeats: $37.85 per month	Beta<br>-<br>-<br>-	[24]<br>[25]<br>[25]<br>[28]
	CAU comparator: Specialised mental healthcare (CAU 2)		
Visit to prepare GP mental health treatment plan (0 months) [b]	Cost of an initial GP visit: $98.67	-	[28]
Allocation of patients to different treatments in specialised mental healthcare (0-8 months)	Medication only: 51.0% (N: 158)<br>Psychotherapy only: 25.8% (N: 80)<br>Combination of both: 23.2% (N: 72)	Dirichlet	[26]
Medication algorithm for depression (0-8 months)	Proportion switching to 2nd-line treatment: 51.6% (SE: 0.8)<br>Cost of 1st-line treatment: $9.91 per month<br>Cost of 2nd-line treatment (post-switch): $17.74 per month<br>GP visits for medication repeats: $37.85 per month	Beta<br>-<br>-<br>-	[24]<br>[25]<br>[25]<br>[28]
Medication algorithm for anxiety (0-8 months)	Proportion switching to 2nd-line treatment: 51.6% (SE: 0.8)<br>Cost of 1st-line treatment: $9.91 per month<br>Cost of 2nd-line treatment (post-switch): $10.38 per month<br>GP visits for medication repeats: $37.85 per month	Beta<br>-<br>-<br>-	[24]<br>[25]<br>[25]<br>[28]
Psychologist visits among patients who receive psychotherapy only (0-8 months)	No. of psychotherapy sessions: 6 (range: 8 - 10)<br>Cost per psychologist session: $106.75	Pert<br>-	Assumption<br>[28]
Psychiatrist visits among patients who receive combination therapy (0-8 months)	No. of psychotherapy sessions: 6 (range: 8 - 10)<br>Cost per psychiatrist session: $164.39	Pert<br>-	Assumption<br>[28]

Parameter	Value and uncertainty range (when applicable)	Uncertainty distribution	Sources
	CAU comparator: Benzodiazepine use (CAU 9)		
All patients who are prescribed benzodiazepines (0-8 months)	8-month probability of benzodiazepine use: 28.0% (SE: 5.7)<br>Cost of benzodiazepines: $14.58 per month	Beta<br>-	[17]<br>[25]
	Other cost parameters		
Annual cost of treatment per case of depression / anxiety (cost offsets)	$838.64 (SE: 1,014.27)	Lognormal	[29]
Cost per patient hour (patient time & travel)	$38.22 (range: ±20%)	Pert	[30]
Cost per patient trip (patient time & travel)	$15.43 (range: ±20%)	Pert	[30]

Abbreviations: CAU - care-as-usual; CSC - collaborative stepped care; GP - general practitioner or general practice; MH - mental health; no. - number; SE - standard error.

[a] *Wage rates were adjusted to incorporate 30% on-costs - i.e., additional loadings to account for administration costs, leave, superannuation, etc.*

[b] *In Australia, commencement of mental health treatment in the formal mental healthcare system always begins with an initial GP consult to develop a GP mental health care plan. This applies to all patients who begin mental health treatment at 0 months (baseline).*

Table S8. Time and travel cost calculations

Intervention step and line item	Description of time and travel costs
CSC 1a: Guided self-help only (Time point: 0-4 months)	
Dep: Visit to prepare GP mental health treatment plan	Each patient had a single consult with a GP which lasted 40 minutes. Each consult involved 2 trips per patient.
Dep: Guided self-help over 4-month period	Each patient had five sessions lasting 45 minutes each with a GP mental health nurse. Each session involved 2 trips per patient.
Anx: Visit to prepare GP mental health treatment plan	Each patient had a single consult with a GP which lasted 40 minutes. Each consult involved 2 trips per patient.
Anx: Guided self-help over 4-month period	Each patient had five sessions lasting 45 minutes each with a GP mental health nurse. Each session involved 2 trips per patient.
CSC 1b: Guided self-help + antidepressants (Time point: 0-4 months)	
Dep: Visit to prepare GP mental health treatment plan	Each patient had a single consult with a GP which lasted 40 minutes. Each consult involved 2 trips per patient.
Dep: Guided self-help over 4-month period	Each patient had five sessions lasting 45 minutes each with a GP mental health nurse. Each session involved 2 trips per patient.
Dep: 4 GP visits to obtain medication repeats	Each patient had four consults with a GP (i.e., one per month) which lasted 20 minutes each. Each consult involved 2 trips per patient.
Anx: Visit to prepare GP mental health treatment plan	Each patient had a single consult with a GP which lasted 40 minutes. Each consult involved 2 trips per patient.
Anx: Guided self-help over 4-month period	Each patient had five sessions lasting 45 minutes each with a GP mental health nurse. Each session involved 2 trips per patient.
Anx: 4 GP visits to obtain medication repeats	Each patient had four consults with a GP (i.e., one per month) which lasted 20 minutes each. Each consult involved 2 trips per patient.
CSC 2a: Severe cases referred to specialised mental healthcare (Time point: 0-8 months)	
Dep: Visit to prepare GP mental health treatment plan	Each patient had a single consult with a GP which lasted 40 minutes. Each consult involved 2 trips per patient.
Dep – AD only: 8 GP visits to obtain medication repeats	Each patient had eight consults with a GP (i.e., one per month) which lasted 20 minutes each. Each consult involved 2 trips per patient.
Dep – PSY only: 8 psychotherapy sessions with a psychologist	Each patient had eight consults with a psychologist (range: 6 to 10) which lasted 50 minutes each. Each consult involved 2 trips per patient.
Dep – AD+PSY: 8 GP visits to obtain medication repeats	Each patient had eight consults with a GP (i.e., one per month) which lasted 20 minutes each. Each consult involved 2 trips per patient.
Dep – AD+PSY: 8 psychotherapy sessions with a psychiatrist	Each patient had eight consults with a psychiatrist (range: 6 to 10) which lasted 50 minutes each. Each consult involved 2 trips per patient.
Anx: Visit to prepare GP mental health treatment plan	Each patient had a single consult with a GP which lasted 40 minutes. Each consult involved 2 trips per patient.
Anx – AD only: 8 GP visits to obtain medication repeats	Each patient had eight consults with a GP (i.e., one per month) which lasted 20 minutes each. Each consult involved 2 trips per patient.
Anx – PSY only: 8 psychotherapy sessions with a psychologist	Each patient had eight consults with a psychologist (range: 6 to 10) which lasted 50 minutes each. Each consult involved 2 trips per patient.

Intervention step and line item	Description of time and travel costs
Anx – AD+PSY: 8 GP visits to obtain medication repeats	Each patient had eight consults with a GP (i.e., one per month) which lasted 20 minutes each. Each consult involved 2 trips per patient.
Anx – AD+PSY: 8 psychotherapy sessions with a psychiatrist	Each patient had eight consults with a psychiatrist (range: 6 to 10) which lasted 50 minutes each. Each consult involved 2 trips per patient.
CSC 2b: Non-responders referred to specialised mental healthcare (Time point: 4-8 months)	
Dep: Visit to prepare GP mental health treatment plan	Each patient had a single consult with a GP which lasted 40 minutes. Each consult involved 2 trips per patient.
Dep – AD only: 4 GP visits to obtain medication repeats	Each patient had four consults with a GP (i.e., one per month) which lasted 20 minutes each. Each consult involved 2 trips per patient.
Dep – PSY only: 8 psychotherapy sessions with a psychologist	Each patient had eight consults with a psychologist (range: 6 to 10) which lasted 50 minutes each. Each consult involved 2 trips per patient.
Dep – AD+PSY: 4 GP visits to obtain medication repeats	Each patient had four consults with a GP (i.e., one per month) which lasted 20 minutes each. Each consult involved 2 trips per patient.
Dep – AD+PSY: 8 psychotherapy sessions with a psychiatrist	Each patient had eight consults with a psychiatrist (range: 6 to 10) which lasted 50 minutes each. Each consult involved 2 trips per patient.
Anx: Visit to prepare GP mental health treatment plan	Each patient had a single consult with a GP which lasted 40 minutes. Each consult involved 2 trips per patient.
Anx – AD only: 4 GP visits to obtain medication repeats	Each patient had four consults with a GP (i.e., one per month) which lasted 20 minutes each. Each consult involved 2 trips per patient.
Anx – PSY only: 8 psychotherapy sessions with a psychologist	Each patient had eight consults with a psychologist (range: 6 to 10) which lasted 50 minutes each. Each consult involved 2 trips per patient.
Anx – AD+PSY: 4 GP visits to obtain medication repeats	Each patient had four consults with a GP (i.e., one per month) which lasted 20 minutes each. Each consult involved 2 trips per patient.
Anx – AD+PSY: 8 psychotherapy sessions with a psychiatrist	Each patient had eight consults with a psychiatrist (range: 6 to 10) which lasted 50 minutes each. Each consult involved 2 trips per patient.
CSC 2c: Remitted cases that relapse & require specialised mental healthcare (Time point: 4-8 months)	
Dep: Visit to prepare GP mental health treatment plan	Each patient had a single consult with a GP which lasted 40 minutes. Each consult involved 2 trips per patient.
Dep – AD only: 4 GP visits to obtain medication repeats	Each patient had four consults with a GP (i.e., one per month) which lasted 20 minutes each. Each consult involved 2 trips per patient.
Dep – PSY only: 8 psychotherapy sessions with a psychologist	Each patient had eight consults with a psychologist (range: 6 to 10) which lasted 50 minutes each. Each consult involved 2 trips per patient.
Dep – AD+PSY: 4 GP visits to obtain medication repeats	Each patient had four consults with a GP (i.e., one per month) which lasted 20 minutes each. Each consult involved 2 trips per patient.
Dep – AD+PSY: 8 psychotherapy sessions with a psychiatrist	Each patient had eight consults with a psychiatrist (range: 6 to 10) which lasted 50 minutes each. Each consult involved 2 trips per patient.
Anx: Visit to prepare GP mental health treatment plan	Each patient had a single consult with a GP which lasted 40 minutes. Each consult involved 2 trips per patient.
Anx – AD only: 4 GP visits to obtain medication repeats	Each patient had four consults with a GP (i.e., one per month) which lasted 20 minutes each. Each consult involved 2 trips per patient.

Intervention step and line item	Description of time and travel costs
Anx – PSY only: 8 psychotherapy sessions with a psychologist	Each patient had eight consults with a psychologist (range: 6 to 10) which lasted 50 minutes each. Each consult involved 2 trips per patient.
Anx – AD+PSY: 4 GP visits to obtain medication repeats	Each patient had four consults with a GP (i.e., one per month) which lasted 20 minutes each. Each consult involved 2 trips per patient.
Anx – AD+PSY: 8 psychotherapy sessions with a psychiatrist	Each patient had eight consults with a psychiatrist (range: 6 to 10) which lasted 50 minutes each. Each consult involved 2 trips per patient.
CAU 1: Treatment with antidepressants only (Time point: 0-8 months)	
Dep: Visit to prepare GP mental health treatment plan	Each patient had a single consult with a GP which lasted 40 minutes. Each consult involved 2 trips per patient.
Dep: 8 GP visits to obtain medication repeats	Each patient had eight consults with a GP (i.e., one per month) which lasted 20 minutes each. Each consult involved 2 trips per patient.
Anx: Visit to prepare GP mental health treatment plan	Each patient had a single consult with a GP which lasted 40 minutes. Each consult involved 2 trips per patient.
Anx: 8 GP visits to obtain medication repeats	Each patient had eight consults with a GP (i.e., one per month) which lasted 20 minutes each. Each consult involved 2 trips per patient.
CAU 2: Referred to specialised mental healthcare (Time point: 0-8 months)	
Dep: Visit to prepare GP mental health treatment plan	Each patient had a single consult with a GP which lasted 40 minutes. Each consult involved 2 trips per patient.
Dep – AD only: 8 GP visits to obtain medication repeats	Each patient had eight consults with a GP (i.e., one per month) which lasted 20 minutes each. Each consult involved 2 trips per patient.
Dep – PSY only: 8 psychotherapy sessions with a psychologist	Each patient had eight consults with a psychologist (range: 6 to 10) which lasted 50 minutes each. Each consult involved 2 trips per patient.
Dep – AD+PSY: 8 GP visits to obtain medication repeats	Each patient had eight consults with a GP (i.e., one per month) which lasted 20 minutes each. Each consult involved 2 trips per patient.
Dep – AD+PSY: 8 psychotherapy sessions with a psychiatrist	Each patient had eight consults with a psychiatrist (range: 6 to 10) which lasted 50 minutes each. Each consult involved 2 trips per patient.
Anx: Visit to prepare GP mental health treatment plan	Each patient had a single consult with a GP which lasted 40 minutes. Each consult involved 2 trips per patient.
Anx – AD only: 8 GP visits to obtain medication repeats	Each patient had eight consults with a GP (i.e., one per month) which lasted 20 minutes each. Each consult involved 2 trips per patient.
Anx – PSY only: 8 psychotherapy sessions with a psychologist	Each patient had eight consults with a psychologist (range: 6 to 10) which lasted 50 minutes each. Each consult involved 2 trips per patient.
Anx – AD+PSY: 8 GP visits to obtain medication repeats	Each patient had eight consults with a GP (i.e., one per month) which lasted 20 minutes each. Each consult involved 2 trips per patient.
Anx – AD+PSY: 8 psychotherapy sessions with a psychiatrist	Each patient had eight consults with a psychiatrist (range: 6 to 10) which lasted 50 minutes each. Each consult involved 2 trips per patient.

Abbreviations: AD - antidepressants only; AD+PSY – antidepressants and psychotherapy combined; Anx - anxiety; CAU - care-as-usual; CSC - collaborative stepped care; Dep - depression; GP - general practitioner or general practice; PSY - psychotherapy only.

Figure S9. Cost-effectiveness plane for the analysis involving depression only

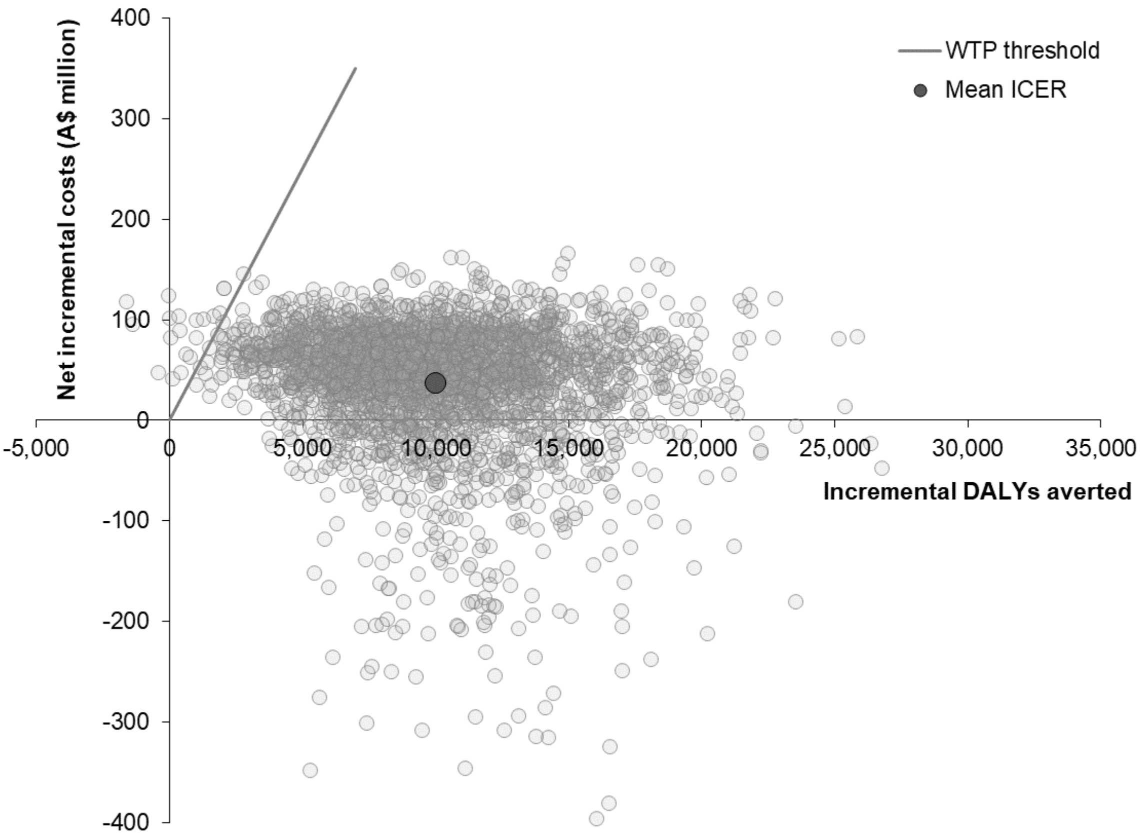

Abbreviations: A$ - Australian dollars; DALYs - disability-adjusted life years; *ICER - incremental cost-effectiveness ratio; WTP - willingness to pay.*

Figure S10. Cost-effectiveness plane for the analysis involving anxiety only

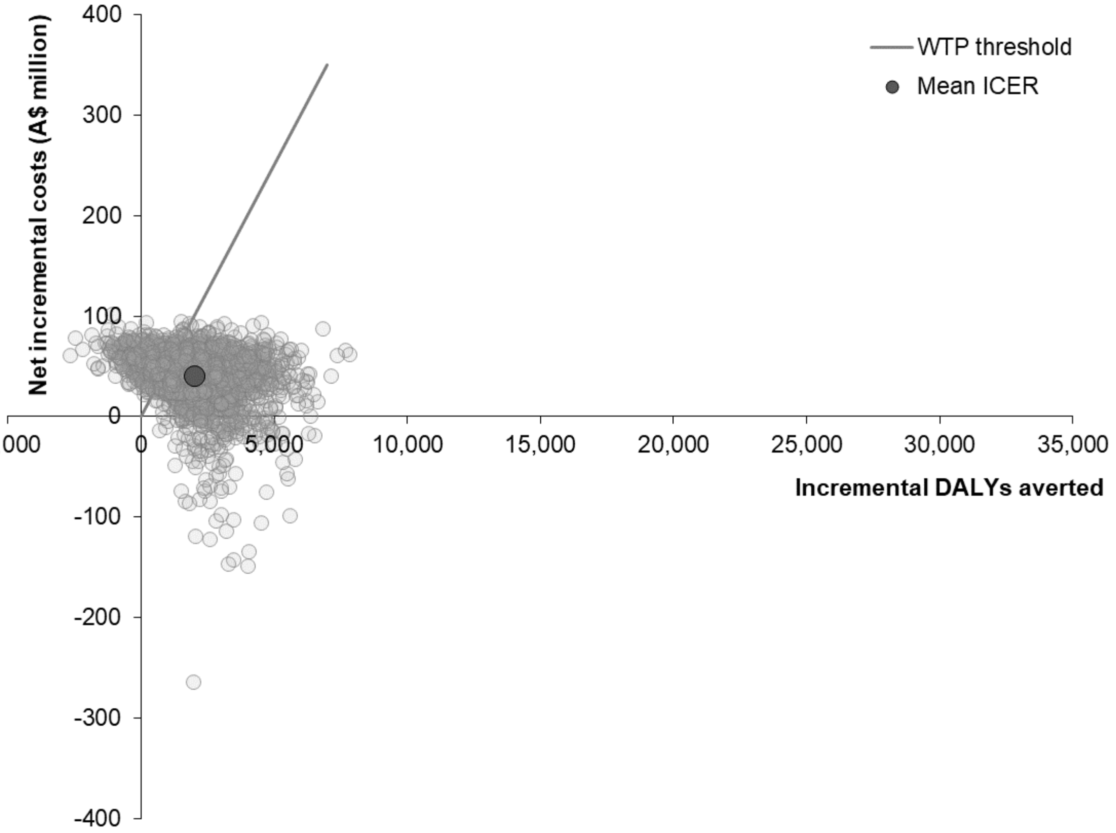

Abbreviations: A$ - Australian dollars; DALYs - disability-adjusted life years; *ICER - incremental cost-effectiveness ratio; WTP - willingness to pay.*

Figure S11. Tornado plot for the univariate sensitivity analysis involving depression and/or anxiety

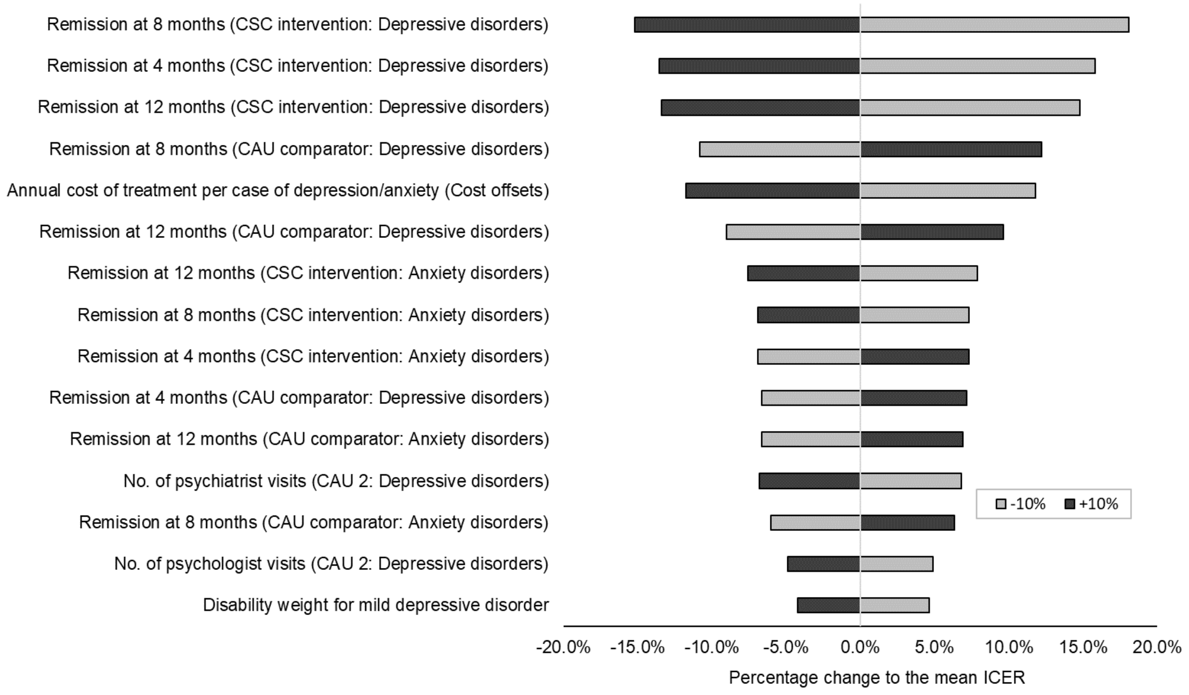

Abbreviations: CAU - care-as-usual; CSC - collaborative stepped care; ICER - incremental cost-effectiveness ratio.

Table S12. Scenario analysis results

Scenario analysis	Depression and/or anxiety<br>*Mean ICER - A$ per DALY averted (95% UI)*	Depression only<br>*Mean ICER - A$ per DALY averted (95% UI)*	Anxiety only<br>*Mean ICER - A$ per DALY averted (95% UI)*
Base case analysis	$5,207 (*dominant* to $25,345)	$3,778 (*dominant* to $23,570)	$20,323 (*dominant* to *dominated*)
SA1. Exclusion of cost offsets	$11,032 ($5,130 to $30,376)	$9,169 ($4,155 to $28,716)	$28,312 ($10,966 to *dominated*)
SA2. Inclusion of time and travel costs	$10,711 (*dominant* to $40,443)	$8,056 (*dominant* to $36,133)	$31,938 (*dominant* to $337,110)

Abbreviations: 95% UI - 95% uncertainty interval; A$ - Australian dollars; DALY - disability-adjusted life years; ICER - incremental cost-effectiveness ratio.

Note: A dominant ICER indicates that the intervention costs less and is more effective than the comparator (i.e., South-East quadrant of the cost-effectiveness plane). Conversely, a dominated ICER indicates that the intervention costs more and is less effective than the comparator (i.e., North-West quadrant of the cost-effectiveness plane).

Reference list

1. Ho FY, Yeung WF, Ng TH, Chan CS. The Efficacy and Cost-Effectiveness of Stepped Care Prevention and Treatment for Depressive and/or Anxiety Disorders: A Systematic Review and Meta-Analysis. Sci Rep 2016;6:29281.

2. van Straten A, Hill J, Richards DA, Cuijpers P. Stepped care treatment delivery for depression: a systematic review and meta-analysis. Psychol Med 2015;45(2):231-46.

3. Apil SR, Hoencamp E, Judith Haffmans PM, Spinhoven P. A stepped care relapse prevention program for depression in older people: a randomized controlled trial. Int J Geriatr Psychiatry 2012;27(6):583-91.

4. Dozeman E, van Marwijk HW, van Schaik DJ, Smit F, Stek ML, van der Horst HE, et al. Contradictory effects for prevention of depression and anxiety in residents in homes for the elderly: a pragmatic randomized controlled trial. Int Psychogeriatr 2012;24(8):1242-51.

5. van't Veer-Tazelaar PJ, van Marwijk HW, van Oppen P, van Hout HP, van der Horst HE, Cuijpers P, et al. Stepped-care prevention of anxiety and depression in late life: a randomized controlled trial. Arch Gen Psychiatry 2009;66(3):297-304.

6. Zhang DX, Lewis G, Araya R, Tang WK, Mak WW, Cheung FM, et al. Prevention of anxiety and depression in Chinese: a randomized clinical trial testing the effectiveness of a stepped care program in primary care. J Affect Disord 2014;169:212-20.

7. Bot M, Pouwer F, Ormel J, Slaets JP, de Jonge P. Predictors of incident major depression in diabetic outpatients with subthreshold depression. Diabet Med 2010;27(11):1295-301.

8. Patel V, Weiss HA, Chowdhary N, Naik S, Pednekar S, Chatterjee S, et al. Effectiveness of an intervention led by lay health counsellors for depressive and anxiety disorders in primary care in Goa, India (MANAS): a cluster randomised controlled trial. Lancet 2010;376(9758):2086-95.

9. Muntingh A, van der Feltz-Cornelis C, van Marwijk H, Spinhoven P, Assendelft W, de Waal M, et al. Effectiveness of collaborative stepped care for anxiety disorders in primary care: a pragmatic cluster randomised controlled trial. Psychother Psychosom 2014;83(1):37-44.

10. Harter M, Watzke B, Daubmann A, Wegscheider K, Konig HH, Brettschneider C, et al. Guideline-based stepped and collaborative care for patients with depression in a cluster-randomised trial. Sci Rep 2018;8(1):9389.

11. Seekles W, van Straten A, Beekman A, van Marwijk H, Cuijpers P. Stepped care treatment for depression and anxiety in primary care. a randomized controlled trial. Trials 2011;12:171.

12. Tolin DF, Diefenbach GJ, Gilliam CM. Stepped care versus standard cognitive-behavioral therapy for obsessive-compulsive disorder: a preliminary study of efficacy and costs. Depress Anxiety 2011;28(4):314-23.

13. Anderson J, Proudfoot J, Gale N, Christensen H, Reeves P, O'Moore K. Implementation and cost effectiveness evaluation of an integrated mental health stepped care service for adults in primary care. Int J Integr Care 2019;19(4):446.

14. IHME. Global Burden of Disease Study 2019 (GBD 2019) Data Resources [Online]. Institute for Health Metrics and Evaluation. 2021 [cited 20 Nov 2020]; Available from: http://ghdx.healthdata.org/gbd-2019

15. Slade T, Johnston A, Oakley Browne MA, Andrews G, Whiteford H. 2007 National Survey of Mental Health and Wellbeing: methods and key findings. Australian New Zealand Journal of Psychiatry 2009;43(7):594-605.

16. ABS. National, state and territory population, June 2020 [Online]. Australian Bureau of Statistics. 2020 [cited 19 Dec 2020]; Available from: https://www.abs.gov.au/statistics/people/population/national-state-and-territory-population/jun-2020

17. Oosterbaan DB, Verbraak MJ, Terluin B, Hoogendoorn AW, Peyrot WJ, Muntingh A, et al. Collaborative stepped care v. care as usual for common mental disorders: 8-month, cluster randomised controlled trial. Br J Psychiatry 2013;203(2):132-9.

18. Burstein R, Fleming T, Haagsma J, Salomon JA, Vos T, Murray CJ. Estimating distributions of health state severity for the global burden of disease study. Population Health Metrics 2015;13:31.

19. Department of Health. Health Workforce Data - Summary Statistics [Online]. Australian Government Department of Health. 2020 [cited 10 Nov 2020]; Available from: https://hwd.health.gov.au/summary.html#part-3

20. RACGP. General Practice: Health of the Nation 2019 [Online]. The Royal Australian College of General Practitioners. 2019 [cited 10 Nov 2020]; Available from: https://www.racgp.org.au/health-of-the-nation/health-of-the-nation

21. ABS. Employee Earnings and Hours, Australia, May 2018 [Online]. Australian Bureau of Statistics. 2019 [cited 16 Nov 2020]; Available from: https://www.abs.gov.au/statistics/labour/earnings-and-work-hours/employee-earnings-and-hours-australia/may-2018

22. Lewinsohn PM, Antonuccio DO, Steinmetz-Breckenridge JL, Teri L. The Coping with Depression course: a psychoeducational intervention for unipolar depression. Eugene OR: Castalia Publishing; 1984.

23. White J. Stresspac: a controlled trial of a self-help package for the anxiety disorders. Behav Cogn Psychother 1995;23(2):89-107.

24. Rush AJ, Trivedi MH, Wisniewski SR, Nierenberg AA, Stewart JW, Warden D, et al. Acute and longer-term outcomes in depressed outpatients requiring one or several treatment steps: a STAR*D report. Am J Psychiatry 2006;163(11):1905-17.

25. Services Australia. Pharmaceutical Benefits Schedule Item Reports [Online]. Commonwealth of Australia. 2021 [cited 10 Nov 2020]; Available from: http://medicarestatistics.humanservices.gov.au/statistics/pbs_item.jsp

26. Gunn JM, Gilchrist GP, Chondros P, Ramp M, Hegarty KL, Blashki GA, et al. Who is identified when screening for depression is undertaken in general practice? Baseline findings from the Diagnosis, Management and Outcomes of Depression in Primary Care (diamond) longitudinal study. Med J Aust 2008;188(12 Suppl):S119-25.

27. Wang PS, Lane M, Olfson M, Pincus HA, Wells KB, Kessler RC. Twelve-month use of mental health services in the United States: results from the National Comorbidity Survey Replication. Arch Gen Psychiatry 2005;62(6):629-40.

28. Services Australia. Medicare Item Reports [Online]. Commonwealth of Australia. 2021 [cited 10 Nov 2020]; Available from: http://medicarestatistics.humanservices.gov.au/statistics/mbs_item.jsp

29. Lee YC, Chatterton ML, Magnus A, Mohebbi M, Le LK, Mihalopoulos C. Cost of high prevalence mental disorders: Findings from the 2007 Australian National Survey of Mental Health and Wellbeing. Aust N Z J Psychiatry 2017;51(12):1198-211.

30. Vos T, Carter R, Barendregt JJ, Mihalopoulos C, Veerman JL, Magnus A, et al. Assessing Cost-Effectiveness in Prevention (ACE–Prevention): Final Report. Brisbane: University of Queensland; 2010.
